# Loss of SREBP-1c ameliorates iron-induced liver fibrosis by decreasing lipocalin-2

**DOI:** 10.1038/s12276-024-01213-2

**Published:** 2024-04-16

**Authors:** Eun-Ho Lee, Jae-Ho Lee, Do-Young Kim, Young-Seung Lee, Yunju Jo, Tam Dao, Kyung Eun Kim, Dae-Kyu Song, Ji Hae Seo, Young-Kyo Seo, Je Kyung Seong, Changjong Moon, Eugene Han, Mi Kyung Kim, Seungwan Ryu, Minsang Shin, Gu Seob Roh, Hye Ra Jung, Timothy F. Osborne, Dongryeol Ryu, Tae-Il Jeon, Seung-Soon Im

**Affiliations:** 1 Department of Physiology, Keimyung University School of Medicine, Daegu, 42601 Republic of Korea; 2 Department of Animal Science, College of Agriculture and Life Science, Chonnam National University, Gwangju, 61186 Republic of Korea; 3 Department of Biomedical Science and Engineering, Gwangju Institute of Science and Technology (GIST), Gwangju, 61005 Republic of Korea; 4 Department of Molecular Cell Biology, Sungkyunkwan University (SKKU) School of Medicine, Suwon, 16419 Republic of Korea; 5 Department of Anatomy, College of Medicine, Institute of Medical Science, Gyeongsang National University, Jinju, 52727 Republic of Korea; 6 Department of Biochemistry, School of Medicine, Keimyung University, Daegu, 42601 Republic of Korea; 7 Aging Research Center, Korea Research Institute of Bioscience and Biotechnology, Daejeon, 34141 Republic of Korea; 8 Laboratory of Developmental Biology and Genomics, Research Institute for Veterinary Science, BK21 PLUS Program for Creative Veterinary Science Research, College of Veterinary Medicine, Seoul National University, Seoul, 08826 Republic of Korea; 9 Department of Veterinary Anatomy and Animal Behavior, College of Veterinary Medicine and BK21 FOUR Program, Chonnam National University, Gwangju, 61186 Republic of Korea; 10 Department of Internal Medicine, Keimyung University School of Medicine, Daegu, 42601 Republic of Korea; 11 Department of Surgery, Keimyung University School of Medicine, Daegu, 42601 Republic of Korea; 12 Department of Microbiology, School of Medicine, Kyungpook National University, Daegu, 42601 Republic of Korea; 13 Department of Pathology, Keimyung University School of Medicine, Daegu, 42601 Republic of Korea; 14 Institute for Fundamental Biomedical Research, Department of Medicine and Biological Chemistry, Johns Hopkins University School of Medicine, St. Petersburg, FL 33701 USA

**Keywords:** Obesity, Metabolic syndrome

## Abstract

Sterol regulatory element-binding protein (SREBP)-1c is involved in cellular lipid homeostasis and cholesterol biosynthesis and is highly increased in nonalcoholic steatohepatitis (NASH). However, the molecular mechanism by which SREBP-1c regulates hepatic stellate cells (HSCs) activation in NASH animal models and patients have not been fully elucidated. In this study, we examined the role of SREBP-1c in NASH and the regulation of LCN2 gene expression. Wild-type and SREBP-1c knockout (1cKO) mice were fed a high-fat/high-sucrose diet, treated with carbon tetrachloride (CCl_4_), and subjected to lipocalin-2 (LCN2) overexpression. The role of LCN2 in NASH progression was assessed using mouse primary hepatocytes, Kupffer cells, and HSCs. LCN2 expression was examined in samples from normal patients and those with NASH. LCN2 gene expression and secretion increased in CCl_4_-induced liver fibrosis mice model, and SREBP-1c regulated LCN2 gene transcription. Moreover, treatment with holo-LCN2 stimulated intracellular iron accumulation and fibrosis-related gene expression in mouse primary HSCs, but these effects were not observed in 1cKO HSCs, indicating that SREBP-1c-induced LCN2 expression and secretion could stimulate HSCs activation through iron accumulation. Furthermore, LCN2 expression was strongly correlated with inflammation and fibrosis in patients with NASH. Our findings indicate that SREBP-1c regulates *Lcn2* gene expression, contributing to diet-induced NASH. Reduced *Lcn2* expression in 1cKO mice protects against NASH development. Therefore, the activation of *Lcn2* by SREBP-1c establishes a new connection between iron and lipid metabolism, affecting inflammation and HSCs activation. These findings may lead to new therapeutic strategies for NASH.

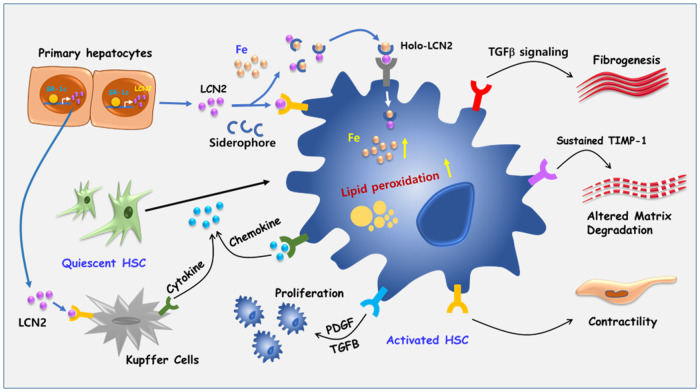

## SREBP-1c deficiency ameliorates iron-induced liver fibrosis through decrease of LCN2

Sterol regulatory element-binding protein (SREBP)-1c plays a role in liver fat regulation and is elevated in nonalcoholic steatohepatitis (NASH). Researchers investigated role of SREBP-1c in NASH using mice models and patient samples, focusing on lipocalin-2 (LCN2) regulation. In NASH mice, LCN2 expression was increased, driven by SREBP-1c. LCN2 treatment increased iron accumulation and fibrosis-related gene expression in hepatic stellate cells, dependent on SREBP-1c. LCN2 expression correlated with inflammation and fibrosis in NASH patients. Reduced LCN2 expression protected against NASH in mice lacking SREBP-1c. These findings highlight role of SREBP-1c in NASH via LCN2, revealing a link between iron and lipid metabolism, potentially suggesting new NASH therapy.

This summary was initially drafted using artificial intelligence, then revised and fact-checked by the author.

## Introduction

Nonalcoholic steatohepatitis (NASH) is a progressive form of nonalcoholic fatty liver disease (NAFLD) and is characterized by the accumulation of fat in hepatocytes, increased immune cell activation, increased inflammation, and the activation of hepatic stellate cells (HSCs), all of which can lead to liver fibrosis^1,2^. Crosstalk between HSCs and other liver cell types is crucial for maintaining liver homeostasis^3^. Dysregulation of hepatocyte lipid metabolism and lipotoxicity can influence HSCs activation in NASH^4,5^. Under disease conditions, HSCs undergo transdifferentiation from quiescent cells to myofibroblast-like cells which are the primary sources of extracellular matrix proteins during liver injury and liver fibrosis. Fat accumulation in hepatocytes is considered the initial step; however, it is insufficient to induce hepatic inflammation and activate HSCs during NASH development^6,7^. In fact, hepatic lipid accumulation alone is a relatively benign condition, 15% of patients progress to NASH. However, the mechanisms involved in the progression from fat accumulation in hepatocytes to activation of immune cells and HSCs have not been identified.

Sterol regulatory element-binding protein (SREBP)-1c is a dominant hepatic isoform involved in lipid accumulation^8^. SREBP-1c plays a predominant role in activating fatty acid (FA) accumulation by binding to the promoters of target genes, followed by transcriptional activation^9^. Over the past four decades, numerous targets of SREBP-1c have been identified in the liver and other tissues^10^. A previous study indicated that SREBP-1c was involved in lipid catabolism by regulating autophagy, a cellular process in high-fat diet (HFD)-induced NAFLD^11^. Activation of de novo lipogenesis by SREBP-1c is upregulated in NAFLD; thus, SREBP-1c has been recognized as an attractive therapeutic target for NAFLD treatment.

Lipocalin-2 (LCN2), which is also known as neutrophil gelatinase-associated lipocalin, is expressed in various organs and tissues, including the brain, bone, heart, kidney, liver, and lung^12^. It acts as a mediator of metabolic inflammation^13^ and is associated with the pathogenesis of diseases such as acute kidney injury, diabetes, obesity, cancer, and cardiovascular disease^14^. LCN2 is known to bind to matrix metalloproteinases (MMPs) and small hydrophobic molecules, forming complexes with membrane receptors in vivo^14,15^. Although the exact endogenous ligand remains unknown^16^, LCN2 is secreted by neutrophils and other cell types, such as hepatocytes and intestinal epithelial cells, and it activates inflammatory pathways in hepatocytes^17,18^. It plays a role in iron transport and immune responses and exerts bactericidal effects by sequestering iron-containing siderophores^19–22^. Increased levels of LCN2 in metabolic disease are linked to cardiovascular mortality, suggesting its potential as a diagnostic and predictive marker^23^. The unique characteristics of LCN2 make it an intriguing protein within the larger lipocalin family, but further research is needed to fully understand its functions and mechanisms in various physiological and pathological contexts.

Additionally, studies indicate that normal iron metabolism is disrupted in ~30% of patients with NAFLD^24^. In this study, we investigated the role of SREBP-1c in regulating LCN2 expression in the liver, which contributes to iron-induced liver fibrosis and NASH.

## Methods

### Human participants

All procedures were approved by the Institutional Review Board of Keimyung University Dongsan Medical Center in Korea (IRB No. DSMC 2022-03-011), and written informed consent was obtained from all participants. Human serum and liver tissues from patients with NASH at various stages were obtained from Keimyung University Dongsan Medical Center Biobank. Based on the patient dataset provided by human biobank at Dongsan Medical Center, we calculated the average and median ranges for normal patients (*n* = 36) and NASH patients (*n* = 35) to represent the physical and biochemical parameters, as shown in Supplementary Table 1.

### Animal experiments

Male wildtype (WT) and SREBP-1c knockout (1cKO) mice were randomly assigned to groups and were fed a normal chow diet (OrientBio, South Korea) or a high-fat/high-sucrose diet (HFHS, D12327, 40% fat, and sucrose as kcal, Research Diets, NJ, USA) for 20 weeks (*n* = 5–10 per group). To assess liver fibrosis, 8 week-old mice received intramuscular injections of 2 mL/kg of carbon tetrachloride (CCl_4_, Sigma‒Aldrich, St. Louis. MO, USA) dissolved in olive oil twice per week for 5 weeks (*n* = 3–5 per group). Control mice received an equal volume of olive oil. CCl_4_-treated mice were further divided into the following experimental groups: CCl_4_ WT, CCl_4_ 1cKO, and CCl_4_+ad-LCN2 1cKO. Additionally, WT and 1cKO mice were administered with CCl_4_ (2 mL/kg body weight) injection to investigate the association between LCN2 and liver fibrosis. To overexpress LCN2, 1cKO mice were intravenously injected with ad-LCN2 (4 × 10^9^ IFU/mL) once, and control mice were injected with equal volumes of GFP. All mice were housed in ventilated cages at a constant temperature (23 °C) under a 12:12 h light-dark cycle (6 a.m.– 6 p.m. light, 6 p.m.– 6 a.m. dark) in a specific pathogen-free facility. All animal experiments complied with the Institutional Animal Care and Use Committee (IRB No. KM-2022-25R1) of Keimyung University in Daegu, Republic of Korea.

### Statistical analysis

The data are presented as the mean ± SEM. A two-tailed, unpaired Student’s *t*-test was used for pairwise comparisons. One-way ANOVA and two-way ANOVA followed by Tukey’s multiple comparisons test were used when comparing three or more groups, as described in the figure legends. Statistical analysis was performed using GraphPad Prism 9.5.1 software (San Diego, CA, USA). Differences were considered significant at *p* < 0.05.

## Results

### Severe HFHS-induced NASH development was prevented in SREBP-1c KO mice

**Fig. 1:**
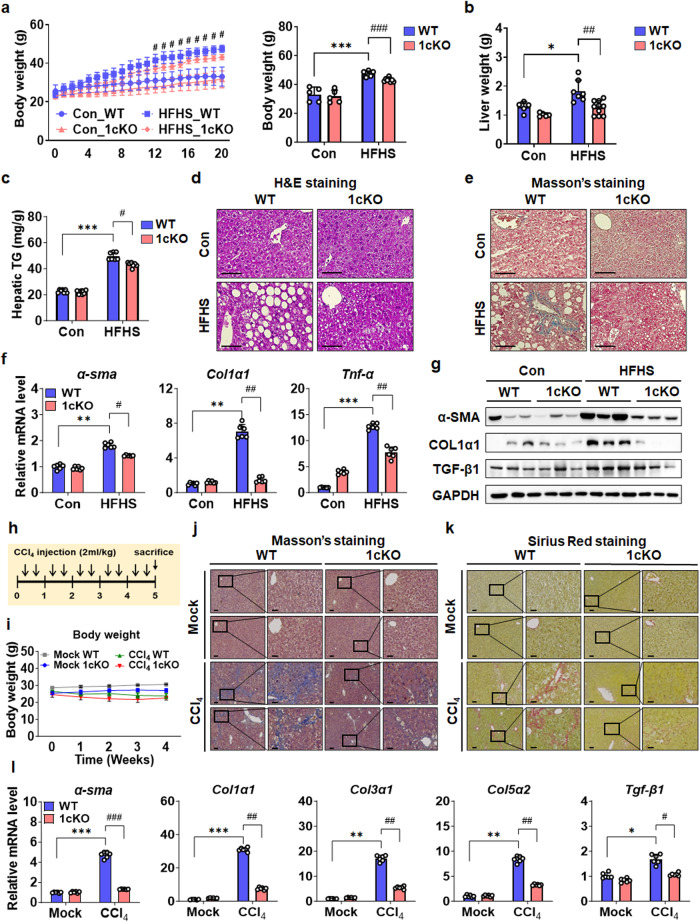
Increased hepatic fibrosis is reduced in 1cKO mice. **a** WT and 1cKO mice were fed a HFHS diet for 20 weeks, to increase body weight (*n* = 5–10 per group). **b** Images of the livers of HFHS-fed mice. **c** Hepatic TG levels in HFHS-fed WT and 1cKO mice. Images of liver sections stained with H&E (**d**) and Masson’s trichrome (**e**) (scale bars: 30 μm). **f** qPCR analysis of *α-sma*, *Col1α1*, and *Tnf-α* mRNA expression in the liver. **g** Immunoblot analysis of α-SMA, COL1α1, and TGF-β1. **h** Scheme for mouse experimental design and CCl_4_ injection. The mice were intramuscularly injected with olive oil or CCl_4_ twice per week for 5 weeks. **i** Body weights of CCl_4_-treated WT and 1cKO mice. Images of Masson’s trichrome (**j**) and Sirius red (**k**) stained liver sections (*n* = 3–5 per group) from CCl_4_-treated WT and 1cKO mice (scale bars: 100 and 30 μm). **l** mRNA levels of *α-sma*, *Col1α1*, *Col3α1*, *Col5α2*, and *Tgf-β1* were measured by qPCR. Values are expressed as the mean ± SEM. *p < 0.05, **p < 0.01, and ***p < 0.001 compared to Con WT mice. ^#^*p* < 0.05 and ^##^*p* < 0.01 compared to HFHS-fed and CCl_4_-treated WT mice.

SREBP-1c deficiency in mice decreased lipogenesis in the liver, and a previous study demonstrated that 1cKO mice were protected from HFD-induced NAFLD^11^. To extend these studies to a diet-induced model of NASH, we fed mice a HFHS diet to induce liver damage that more accurately reflects NASH development in humans. WT mice had increased body weight due to the HFHS diet without a decrease in food intake, as expected (Fig. 1a and Supplementary Fig. 1a), whereas the HFHS-fed 1cKO mice gained less body weight. This was due to decreased fat mass (Supplementary Fig. 1b). Simultaneously, lean body mass increased in 1cKO mice (Supplementary Fig. 1c). Additionally, plasma levels of aspartate aminotransferase (AST), alanine aminotransferase (ALT), triglyceride (TG), and total cholesterol (TC) were determined. AST, ALT, TG, and TC levels were lower in 1cKO mice than in WT mice (Supplementary Fig. 1d). WT mice also had higher hepatic TG levels and total liver weights than 1cKO mice (Fig. 1b, c). These findings were consistent with the H&E staining results (Fig. 1d). The expected increase in lipogenic gene and protein expression induced by HFHS diet in WT was lower in the 1cKO mice (Supplementary Fig. 1e, f). Interestingly, Masson’s trichrome staining revealed significant hepatic fibrosis in HFHS-fed WT mice, and fibrosis was significantly reduced in 1cKO mice (Fig. 1e). Fibrogenic gene and protein levels in whole-liver samples were also lower in 1cKO mice than in HFHS-fed WT mice (Fig. 1f, g and Supplementary Fig. 1g-k). These results indicated that SREBP-1c deficiency protected mice from diet-induced hepatic fibrosis and lipid accumulation.

### CCl_4_-induced liver fibrosis is reduced in SREBP-1c KO mice

NASH is characterized by the combination of excessive hepatic fat accumulation and inflammation that are partly caused by abnormal FA metabolism^2^. However, previous studies have shown that methionine/choline-deficient diets or high-cholesterol diets induce fibrosis and lipid accumulation in the liver^25^. Therefore, to determine whether a NASH-induced target was regulated by SREBP-1c independent of lipid accumulation, we first evaluated gene expression following liver damage induced by acute CCl_4_ administration, which is a robust model of liver fibrosis with a reduced effect on lipid accumulation. To induce NASH, WT and 1cKO mice were injected with CCl_4_ and analyzed after 5 weeks (Fig. 1h). There were no differences in fat mass or lean body mass (Supplementary Fig. 2a, b). Whole-body and total liver weights were decreased by CCl_4_ (Fig. 1i and Supplementary Fig. 2c). Liver fibrosis was confirmed using Masson’s trichrome and sirius red staining, and WT mice exhibited increased fibrosis compared to 1cKO mice (Fig. 1j, k). Histologically, lipid accumulation was slightly increased in the liver samples of WT mice after CCl_4_ administration (Supplementary Fig. 2d). AST and ALT levels were also lower in 1cKO mice than in WT mice (Supplementary Fig. 2e). Interestingly, after the administration of CCl_4,_ the expression levels of the lipogenic factors such as fatty acid synthase (*Fas)* and *Srebp-1c* were suppressed compared to those in the control group (Supplementary Fig. 2f). However, a slight increase in the protein level of the processed form of nSREBP-1 was observed (Supplementary Fig. 2 h), which could be the possible reason for the induction of LCN2 after CCl_4_ administration. Furthermore, the expression of genes related to fibrogenesis and inflammation was increased in the livers of CCl_4_-treated mice in the WT group but not in 1cKO mice group (Fig. 1l and Supplementary Fig. 2g–i). Consistent with previous studies, the expression of genes related to inflammation correlated with increased fibrosis. Taken together, these results indicate that SREBP-1c might be involved in hepatic fibrosis without increasing lipogenic gene expression.

### LCN2 is a novel target of SREBP-1c in the liver

**Fig. 2:**
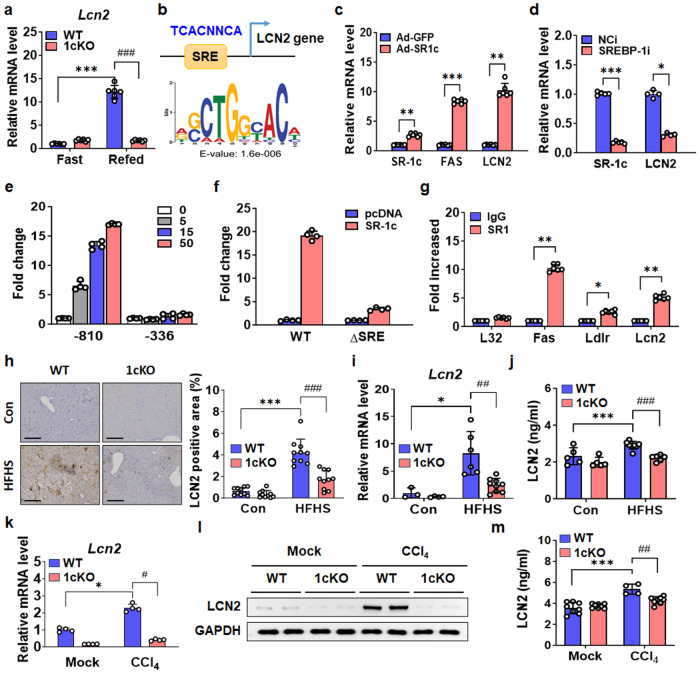
LCN2 expression is regulated by SREBP-1c. **a** *Lcn2* mRNA expression levels in the livers of WT and 1cKO mice were measured after 24 h of fasting followed by 12 h of refeeding. **b** Consensus SREBP-1c binding sequences for LCN2 were identified by ChIP-sequencing and MEME analysis. **c** mRNA levels of *SR-1c*, *FAS*, and *LCN2* in Huh7 cells infected with Ad-SR1c. **d** *SR-1c* and *LCN2* mRNA levels in Huh7 cells transfected with siRNA targeting SREBP-1i. **e** The effect of SREBP-1c on mouse *Lcn2* promoter activity was determined by cotransfecting HEK293T cells with an LCN2 reporter construct and SREBP-1c expression vector. **f** Mutations in the SRE motif of the -829 *Lcn2* reporter were evaluated to determine their impact on reporter activity. **g** ChIP assay was performed to assess SREBP-1 binding in hepatic chromatin from WT or 1cKO mice, and the results were analyzed by qPCR. **h** Immunohistochemical staining of liver sections for LCN2 and quantification of LCN2-positive areas (*n* = 10 per group; scale bars: 30 μm). **i** mRNA expression levels of *Lcn2* in WT and 1cKO mice fed a HFHS diet for 20 weeks. **j** Analysis of serum LCN2 concentrations. **k** qPCR analysis of *Lcn2* gene expression in the livers of WT and 1cKO mice treated with CCl_4_ for 5 weeks. **l** Immunoblot analysis of LCN2 protein levels in the livers of WT and 1cKO mice. **m** Measurement of serum LCN2 concentrations. Values represent mean ± SEM. *p < 0.05, **p < 0.01, and ***p < 0.001 compared to respective control groups. ^##^*p* < 0.01 and ^###^*p* < 0.001 compared to corresponding WT mice.

SREBP-1c expression is strongly induced by refeeding status^26^; therefore, we performed RNA microarray analysis utilizing the livers of fasted/refed WT mice to identify SREBP-1c targets^27^. *Lcn2* mRNA level was confirmed using quantitative PCR (qPCR) (Fig. 2a), and the result suggested that LCN2 is possibly a novel target of SREBP-1c in the liver. Chromatin immunoprecipitation (ChIP)-sequencing analysis of LCN2 revealed SREBP-1 response element (SRE) peaks in the promoter region^27^. SRE was investigated using consensus SREBP-1c binding sequences on the *LCN2* promoter, and we discovered a highly conserved SRE on *LCN2* gene promoter (Fig. 2b). *LCN2* expression was increased by ectopic SREBP-1c expression mediated by an adenoviral vector (ad-SR1c) (Fig. 2c). Furthermore, *LCN2* expression was also reduced by SREBP-1 inhibition (Fig. 2d). Promoter activity analyzed by luciferase assay was increased in a dose-dependent manner, indicating that SREBP-1c could stimulate *Lcn2* promoter activity (Fig. 2e). In addition, *Lcn2* promoter activity was strongly stimulated by SREBP-1c overexpression, and this activity was decreased by deleting the SRE region from *Lcn2* gene promoter (Fig. 2f). ChIP assays showed that SREBP-1c could possibly bind to the *Lcn2* gene promoter (Fig. 2g). Immunohistochemical staining for LCN2 showed that the LCN2-positive area was increased in WT mice fed with HFHS diet and was decreased in 1cKO mice (Fig. 2h). This finding was consistent with *Lcn2* gene expression data (Fig. 2i). Importantly, circulating levels of LCN2 were also increased in the serum of WT mice fed with HFHS diet, and these levels were lower in the 1cKO group (Fig. 2j). We repeated these measurements in WT and 1cKO mice treated with CCl_4_. Similarly, LCN2 expression was induced by CCl_4_ in WT mice but not in 1cKO mice (Fig. 2k, l). Furthermore, LCN2 activity was increased in the serum of CCl_4_-treated WT mice but was not increased in the serum of 1cKO mice (Fig. 2m). Taken together, these results suggest that SREBP-1c drives liver fibrosis and that LCN2 is a direct target of SREBP-1c.

### LCN2 is mainly expressed and secreted by hepatocytes

**Fig. 3:**
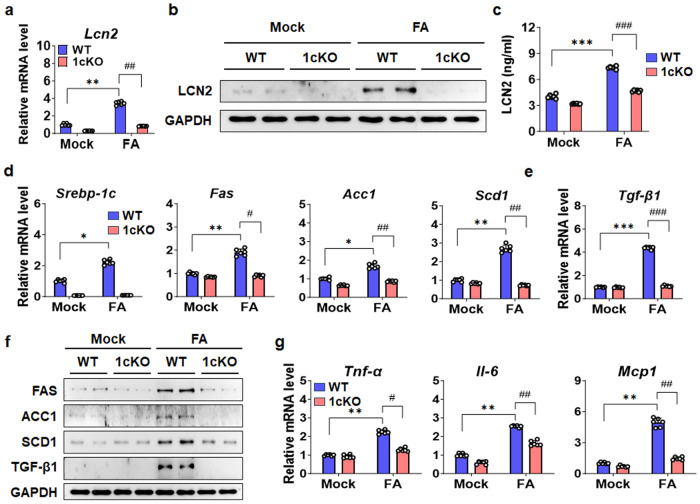
Effect of FA on LCN2 gene expression in primary hepatocytes. **a** *Lcn2* mRNA expression levels in mouse primary hepatocytes after FA treatment for 24 h. **b** LCN2 protein levels in FA-treated mouse primary hepatocytes. **c** LCN2 concentration in the FA treated supernatant medium. **d** Expression levels of the lipogenic genes *Srebp-1c*, *Fas*, *Acc1*, and *Scd1* in FA-treated mouse primary hepatocytes. **e** mRNA expression of *Tgf-β1*. **f** FAS, ACC1, SCD1, and TGF-β1 protein levels in mouse primary hepatocytes. **g** qPCR analysis of *Tnf-α*, *Il-6*, and *Mcp1* mRNA expression in mouse primary hepatocytes after FA treatment. Values are expressed as mean ± SEM. *p < 0.05, **p < 0.01, and ***p < 0.001 compared to Mock WT mice. ^#^*p* < 0.05, ^##^*p* < 0.01, and ^###^*p* < 0.001 compared to FA-treated WT mice.

**Fig. 4:**
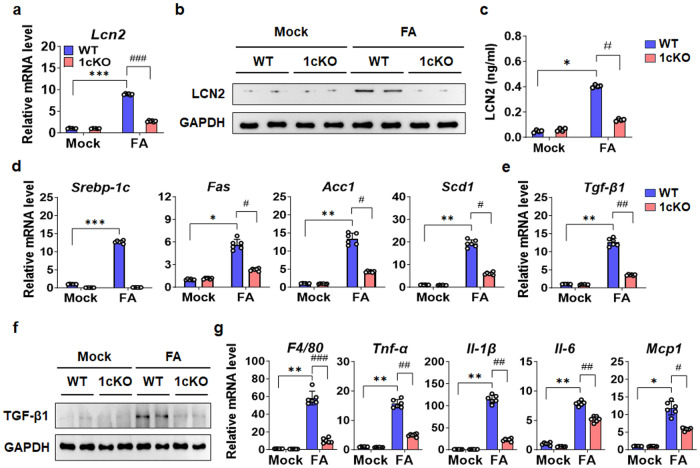
Effect of FA on LCN2 gene expression in KCs. **a** *Lcn2* mRNA expression levels in WT and 1cKO KCs after FA treatment for 24 h. **b** Immunoblot analysis of LCN2 protein levels in KCs. **c** Measurement of LCN2 concentrations in the medium of KCs treated with FA. **d** qPCR analysis of *Srebp-1c*, *Fas*, *Acc1*, and *Scd1* mRNA expression. **e** mRNA expression of *Tgf-β1*. **f** TGF-β1 protein levels in WT and 1cKO KCs treated with FA. **g** *F4/80*, *Tnf-α*, *Il-1β*, *Il-6*, and *Mcp1* mRNA levels in KCs. Values are expressed as mean ± SEM. *p < 0.05, **p < 0.01, and ***p < 0.001 compared to Mock WT mice. ^#^*p* < 0.05, ^##^*p* < 0.01, and ^###^*p* < 0.001 compared to FA-treated WT mice.

Previous studies revealed that LCN2 is expressed and secreted by several cell types, such as hepatocytes, neutrophils, macrophages, and adipocytes ^13,28–31^, and 1cKO mice used in this study is a global KO mice model. Thus, to analyze the potential cellular source that mediates the effects of SREBP-1c on LCN2, we first treated mouse primary hepatocytes with exogenous FA to mimic the HFD. The gene and protein expression of LCN2 was increased by FA treatment but was reduced in 1cKO hepatocytes (Fig. 3a, b). Importantly, the increase in the FA-induced secretion of LCN2 by FA from WT hepatocytes was also blunted in 1cKO hepatocytes (Fig. 3c). The expression of lipogenic targets, such as *Fas*, acetyl-CoA carboxylase 1 (*Acc1*), stearoyl-CoA desaturase 1 (*Scd1*), and transforming growth factor-beta 1 (*Tgf-β1*), in WT hepatocytes was increased by FA treatment, but these effects were significantly inhibited in 1cKO hepatocytes (Fig. 3d, e). A similar pattern of expression was observed in the protein levels (Fig. 3f). As a positive control, we measured tumor necrosis factor alpha (*Tnf-α*), interleukin-6 (*Il-6*), and monocyte chemoattractant protein-1 (*Mcp1*), which are inflammatory markers; the expression levels of these markers were increased by FA treatment in the WT group, and the response was reduced in the 1cKO group (Fig. 3g). Next, LCN2 gene expression and secretion were measured in primary Kupffer cells (KCs) isolated from WT and 1cKO mice. FA treatment slightly increased LCN2 mRNA and protein levels in KCs isolated from WT mice and were reduced in 1cKO KCs (Fig. 4a, b). LCN2 secretion levels were very low in KCs compared with primary hepatocytes (Fig. 4c). The expression of *Fas*, *Acc1*, *Scd1*, and *Tgf-β1*, which are *Srebp-1c* targets was decreased in 1cKO KCs compared to WT KCs (Fig. 4d, e). TGF-β1 protein levels exhibited a similar pattern (Fig. 4f). The gene expression levels of *F4/80*, *Tnf-α*, *Il-1β*, *Il-6*, and *Mcp1* were lower in 1cKO KCs than in WT KCs (Fig. 4g). Similar trends were observed for KCs isolated from WT and 1cKO mice with CCl_4_-induced NASH (Supplementary Fig. 3a–g). In addition, there was no change in the expression of ferroptosis-related genes in hepatocytes isolated from FA-treated WT or 1cKO mice (Supplementary Fig. 4a–c). However, after treatment with FA and knockdown of SREBP-1c by siRNA transfection, the secretion of LCN2 was reduced in human LX-2 HSCs (Supplementary Fig. 4d). These results suggest that LCN2, which is primarily produced by hepatocytes, is highly upregulated under various conditions that induce cellular stress, such as HFD or inflammation.

### LCN2-induced intracellular iron accumulation in primary HSCs

**Fig. 5:**
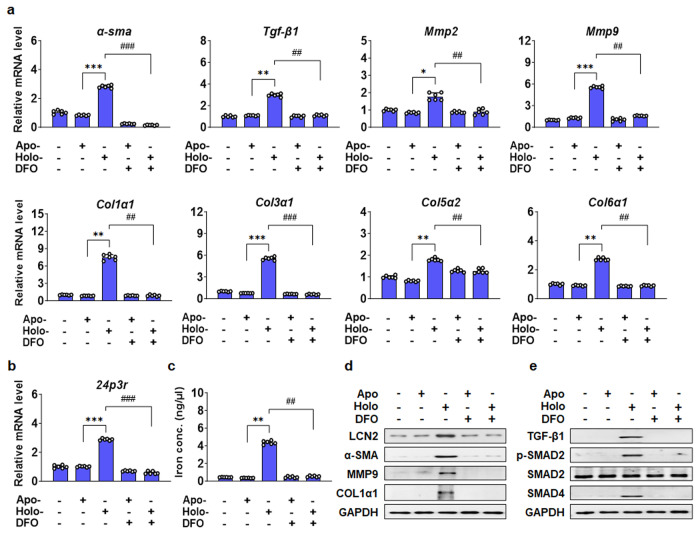
DFO effect on LCN2-induced primary HSCs activation. **a** mRNA expression levels of *α-sma*, *Tgf-β1*, *Mmp2*, *Mmp9*, *Clo1α1*, *Col3α1*, *Col5α2*, and *Col6α1* in primary HSCs treated with apo-LCN2, holo-LCN2, and DFO for 24 h were measured by qPCR. **b** 24p3r gene expression. **c** Iron concentration in the lysates of primary HSCs. **d** Immunoblot analysis of LCN2, α-SMA, MMP9, and COL1α1 protein expression. **e** Protein levels of TGF-β1, *p*-SMAD2, SMAD2, and SMAD4 in primary HSCs treated with apo-LCN2, holo-LCN2, and DFO. Values are expressed as mean ± SEM. *p < 0.05 and **p < 0.01 com*p*ared to mock apo-LCN2-treated primary HSCs. ^#^*p* < 0.05 and ^##^*p* < 0^.^01 compared to DFO-treated *p*rimary HSCs.

Primary HSCs are the major source of extracellular collagen production during liver fibrosis^32^. To examine whether the effects of SREBP-1c on LCN2 were mediated by its effects on HSCs to stimulate fibrogenesis, we treated mouse primary HSCs with either apo-LCN2 or Fe^3+^-conjugated LCN2 (holo-LCN2). Holo-LCN2 but not apo-LCN2 induced expression of several genes known to influence fibrogenesis, including α-smooth muscle actin (*α-sma*), *Tgf-β1*, *Mmp2*, *Mmp9*, collagen type I alpha 1 chain (*Col1α1*), *Col3α1*, *Col5α2*, *Col6α1*, and the Lcn2 receptor protein 24p3 receptor (*24p3r*) (Fig. 5a, b). In contrast, treatment with the iron chelator deferoxamine (DFO) alone had no effect on the expression of these genes, but it completely blocked the effects of holo-LCN2. The increase in cellular iron concentrations following the administration of holo-LCN2 to HSCs was also blocked by the addition of DFO (Fig. 5c). The protein levels of LCN2, *α*-SMA, MMP9, and Col1*α*1 were consistent with the gene expression levels (Fig. 5d). Iron overload activates fibrogenic gene expression via TGF-β signaling in HSCs (Supplementary Fig. 5a). Holo-LCN2 stimulated TGF-β signaling in HSCs, and this effect was sensitive to DFO (Fig. 5e). Changes in intracellular iron have also been shown to alter apoptosis^33^, however, we did not observe any significant changes in the protein levels of poly ADP-ribose polymerase (PARP) or cleaved-PARP (Supplementary Fig. 5b), which are markers of apoptosis. Subsequently, we verified the degree of cell death by annexin V staining (Supplementary Fig. 5c, d).

### Overexpression of LCN2 promoted liver fibrosis in SREBP-1c KO mice

**Fig. 6:**
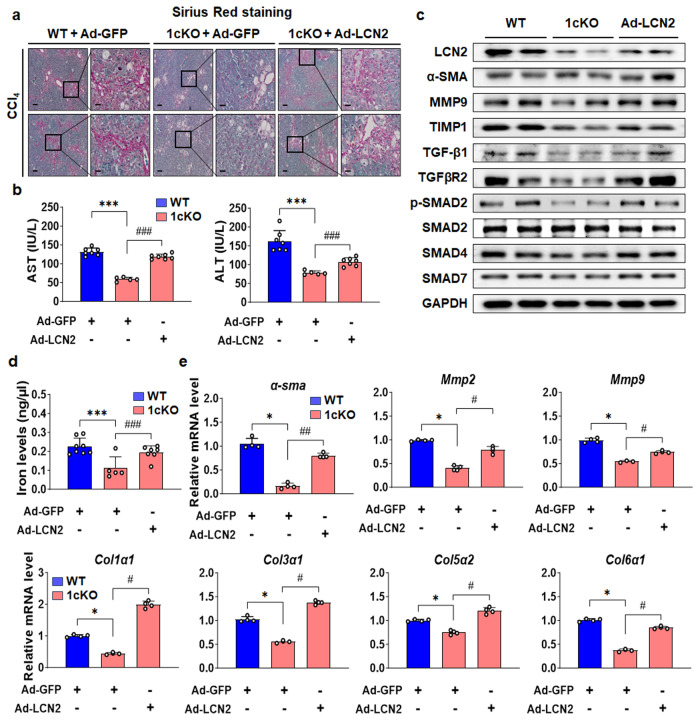
Overexpression of LCN2 in CCl_4_-induced 1cKO mice. **a** Sirius red staining of the livers of CCl_4-_ and ad-LCN2-treated WT and 1cKO mice (*n* = 5–7 per group; scale bars: 100 and 30 μm). **b** Serum AST and ALT levels. **c** LCN2, α-SMA, MMP9, TIMP1, TGF-β1, TGFβR2, p-SMAD2, SMAD2, SMAD4, and SMAD7 protein levels in the liver. **d** Intracellular iron concentration in the liver. **e** ECM and fibrosis marker genes *α-sma, Mmp2*, *Mmp9*, *Col1α1*, *Col3α1*, *Col5α2*, and *Col6α1* in the liver. Values are expressed as mean ± SEM. *p < 0.05 and ***p < 0.001 compared to CCl_4_-treated WT mice. ^#^*p* < 0.05, ^##^*p* < 0.01, and ^###^*p* < 0.001 compared to CCl_4_-treated 1cKO mice.

In a previous study, LCN2 deficiency was shown to protect against HFD‐induced liver fibrosis in *ob/ob* mice^34^. We confirmed that the same phenotype was present in LCN2 KO mice. Histologically, the progression of liver fibrosis was lower in the HFD-fed (20 week-old) LCN2 KO mice than in HFD-fed WT mice, and similar changes in the expression of α-SMA were observed (Supplementary Fig. 6a). The protein level of α-SMA was also lower in the livers of HFD-induced LCN2 KO mice than in HFD-fed WT mice (Supplementary Fig. 6b, c). Thus far, our data support a model in which HFHS increases SREBP-1c-mediated LCN2 activation in hepatocytes and in which the secreted protein drives HSC-mediated liver fibrosis through the direct binding of holo-LCN2 to HSCs. If LCN2 is downstream of SREBP-1c in this pathway, then ectopic expression of LCN2 in 1cKO mice should rescue the development of fibrosis. Figure 6a shows that CCl_4_-dependent Sirius red staining was restored to the level in CCl_4_-treated WT mice when 1cKO mice were injected with an adenovirus designed to express LCN2. Serum levels of AST and ALT showed similar changes (Fig. 6b). Mice body and liver weights were slightly decreased by CCl_4_ injection (Supplementary Fig. 7a, b). The gene expression level of *Srebp-1c* was not restored, as expected (Supplementary Fig. 7c). The protein levels of TGF-β signaling and fibrosis markers in the ad-LCN2-infected 1cKO mice increased to levels similar to those of CCl_4_-treated WT mice (Fig. 6c). Importantly, iron levels in liver tissues were increased in 1cKO mice treated with ad-LCN2 (Fig. 6d). Additionally, the expression levels of fibrosis-related genes and LCN2 target genes, *α-sma*, *Mmp2*, *Mmp9*, *Col1α1*, *Col3α1*, *Col5α2*, *Col6α1*, tissue inhibitor matrix metalloproteinase 1 (*Timp1*), *Tgf-β1*, plasminogen activator inhibitor 1 (*Pai1*), desmin (*Des*), and *24p3r* were increased in the livers of ad-LCN2-infected 1cKO mice (Fig. 6e and Supplementary Fig. 7d-h).

### LCN2 expression is associated with NASH in the human liver

**Fig. 7:**
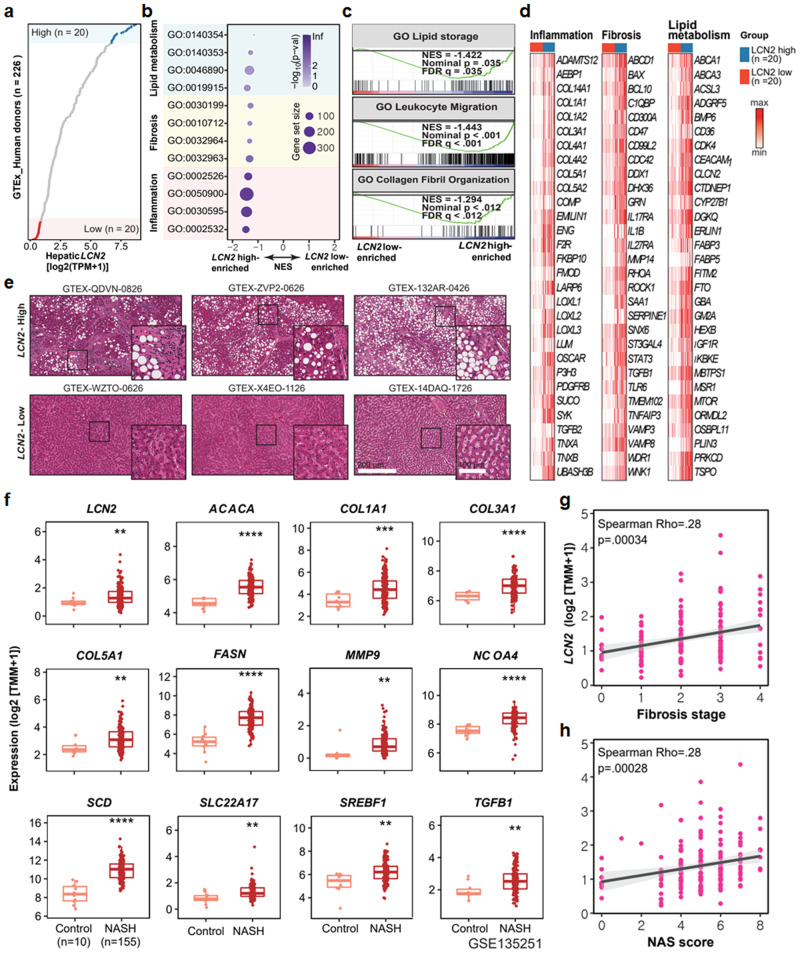
LCN2 expression is associated with lipid metabolism, fibrosis, and inflammation-related genes in the human liver transcriptome. **a** Scatter plot showing hepatic *LCN2* expression in 226 human donors. Bubble plot (**b**), GSEA enrichment plot (**c**), and heatmaps (**d**) showing the GSEA results and the expression profiles of 30 representative genes in 12 gene sets enriched in the LCN2-high subgroup. GSEA was performed on the transcriptomes of the LCN2-high and LCN2-low groups (*n* = 20 per group). **e** H&E-stained liver histologic images from the LCN2-high and LCN2-low groups. The white bars indicate the scales of images. **f** Boxplot showing the expression of each indicated gene involved in NASH. The data are presented as the 25th quartile, the median, and the 75th quartile. Scatter plots illustrating positive correlations between hepatic *LCN2* expression and fibrosis stage (**g**) or NASH score (**h**) in human livers. Values are expressed as mean ± SEM. **p < 0.01, ***p < 0.001, and ****p < 0.0001 compared to control.

Next, we investigated the relationship between hepatic *LCN2* expression and NASH in humans using two independent publicly available datasets: the GTEx and NCBI GEO (GSE135251) datasets^35^. *LCN2* expression in human livers was determined by examining GTEx hepatic transcriptomes (*n* = 226). *LCN2* expression in the liver varied significantly between human donors (Fig. 7a). To determine the effect of *LCN2* expression on fibrosis in human livers, we focused on two groups with the highest and lowest levels of hepatic *LCN2* expression. Approximately 10% of the donors with the highest or lowest *LCN2* expression were assigned to the *LCN2*-high or low group, respectively. We performed GSEA to identify gene sets and cellular pathways associated with hepatic *LCN2* expression. GSEA revealed that gene sets associated with the NASH phenotype were enriched in the *LCN2*-high subgroup (Fig. 7b, c), indicating that hepatic *LCN2* expression was positively correlated with clinically defined NASH in humans. We generated heatmaps to visualize the hepatic expression of 30 representative genes in 12 gene sets in the *LCN2*-high and -low groups (Fig. 7d). The expression of genes involved in lipid metabolism, fibrosis, and inflammation was elevated in the *LCN2*-high group compared to the *LCN2*-low group. Next, we analyzed the H&E-stained results in the GTEx datasets, which revealed that the LCN2-high samples exhibited a NASH-related histological phenotype (Fig. 7e).

**Fig. 8:**
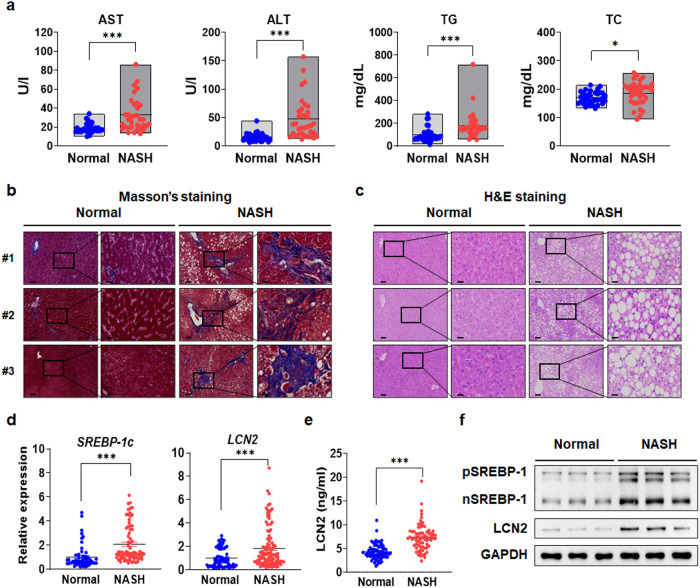
In human patients with NASH livers, LCN2 expression is elevated and positively correlated with NASH phenotypes. **a** AST, ALT, TG, and TC levels in normal and NASH patient serum (normal, *n* = 36; and NASH, *n* = 35). Images of Masson’s trichrome (**b**) and H&E (**c**) stained normal and NASH patient liver sections (scale bars: 100 and 30 μm) (normal, *n* = 6; and NASH, *n* = 35). **d** *SREBP-1c* and *LCN2* mRNA levels (normal, *n* = 26; and NASH, *n* = 35). **e** LCN2 in normal and NASH patient serum (normal, *n* = 26; and NASH, *n* = 35). **f** pSREBP-1, nSREBP-1, and LCN2 protein levels. Values are expressed as mean ± SEM. *p < 0.05 and ***p < 0.001 compared to normal group.

Consistent with the findings in the GTEx dataset, hepatic *LCN2* expression was significantly increased in patients with NASH compared to normal individuals, and these results were similar to the expression patterns of other NASH-associated genes, such as acetyl-CoA carboxylase alpha*, COL1α1, COL3α1, COL5α1, FAS, MMP9*, nuclear receptor coactivator 4*, SCD*, solute carrier family 22 member 17*, SREBF1*, and *TGF-β1* (Fig. 7f). The results from the GTEx dataset indicated that hepatic *LCN2* expression could be linked to the NASH-associated phenotype in humans. In addition to analyzing the GTEx dataset, we evaluated hepatic *LCN2* expression and its association with NAFLD indices, such as the fibrosis score and NAFLD activity score (NAS), in the NAFLD cohort^35^. *LCN2* expression was positively associated with the fibrosis stage (Spearman *Rho* = 0.28, *p* = 0.00034) and NAS (Spearman *Rho* = 0.28, *p* = 0.00028) (Fig. 7g, h). Additionally, plasma AST, ALT, TG, and TC levels were analyzed in normal individuals and NASH patients (*n* = 36), and the levels were higher in the NASH group than in the normal group (Fig. 8a). Physical and biochemical parameters revealed the changes associated with NASH (Supplementary Table 1). Histologically, liver fibrosis was increased in patients with NASH according to Masson’s trichrome staining (Fig. 8b), and hepatic TG levels were consistent with the H&E staining data (Fig. 8c). Similar to the big data analysis, *LCN2* gene expression was significantly increased in patients with NASH; *SREBP-1c* expression and lipogenic gene expression were strongly correlated with *LCN2* gene expression, which was consistent with *Lcn2* activation by SREBP-1c and was similar to the findings of our studies in mice (Fig. 8d). This notion was further supported by the increase in LCN2 secretion in patients with NASH compared to healthy individuals, indicating that LCN2 might be a representative biomarker of NASH development in humans (Fig. 8e). SREBP-1 and LCN2 protein levels were significantly increased in NASH patients (Fig. 8f). Additionally, the expression levels of the fibrosis and inflammatory marker genes, *α-SMA*, *TGF-β1*, *MMP2*, *MMP9*, *COL1α1*, *COL3α1*, *COL5α2*, *COL6α1*, *F4/80*, *TNF-α*, *IL-1β*, and *MCP1* were increased in patients with NASH (Supplementary Fig. 8a, b). Based on our findings from cell and rodent models, these bioinformatic analyses of human liver datasets emphasize the correlation between SREBP-1c, LCN2 and NASH.

## Discussion

LCN2 is a biomarker of various stresses and diseases, including cancer, hepatic steatosis, and acute kidney injury^17^. It plays a role in regulating innate immunity, cell processes, and tumor metastasis^23,30,36^. LCN2 levels are increased in NASH patients and animal models^37,38^. However, the regulatory mechanisms and functions of LCN2 are not fully understood. This study revealed that LCN2 activated HSCs and was increased in NASH mice. Depletion of SREBP-1c attenuated NASH by reducing MMP9/LCN2 signaling in HSCs LCN2-mediated HSCs activation promoted fibrotic gene expression through iron accumulation and the activation of TGF-β signaling.

Iron homeostasis is important for maintaining cell viability and function, and LCN2 is involved in maintaining the viability and function of HSCs^39^. However, disturbances in iron levels can contribute to metabolic diseases^40,41^. Iron promotes HSCs activation through multiple mechanisms. It induces oxidative stress, triggering signaling pathways such as the TGF-β pathway to activate HSCs and stimulate extracellular matrix production^42^. Iron also activates pathways such as the Janus family tyrosine kinase-signal transducers and activators of the transcription pathway, thereby promoting HSC proliferation. Furthermore, iron increases the expression of genes involved in HSCs activation while reducing the expression of those that maintain HSCs quiescence. It can also drive macrophages toward a proinflammatory response, resulting in the release of cytokines and growth factors that enhance HSCs activation^43^. Consequently, the impact of iron on oxidative stress, gene expression, and the immune response plays a significant role in HSCs activation. Therefore, targeting iron metabolism could be a potential therapeutic approach for liver fibrosis treatment.

However, the role of iron reduction in suppressing TGF-β signaling and HSCs activation has not been determined. Iron is known to activate HSCs, which are involved in liver fibrosis. When holo-LCN2 was administered in the absence of TGF-β, fibrogenic markers were activated in HSCs. Previous research has shown that LCN2 promotes liver fibrogenesis and inflammation in NASH patients, and increased plasma LCN2 levels are associated with NASH^37^. Consistent with these findings, *LCN2* expression correlated with liver fibrosis in NASH patients, as revealed by big data analysis.

Furthermore, the iron-chelating molecule DFO suppresses TGF-β signaling in holo-LCN2-induced HSCs by promoting SMAD2 degradation and inhibiting downstream targets^44^. SMAD2 is a crucial component of the TGF-β pathway and is involved in cell processes, including cell growth, differentiation, and apoptosis^45^. Iron chelators, such as the cyclin-dependent kinase inhibitor p21, modulate SMAD2 function and its downstream gene expressions^46^. These findings have implications for the treatment of fibrosis and cancer. SREBP-1 is reported to be involved in the modulation of lipid metabolism in *C. elegans* via zinc-mediated regulation of the SREBP-SCD axis^47^, suggesting a link between SREBP1c and intracellular iron levels. SREBP-1a/1c activates hepcidin expression, regulating systemic iron homeostasis^48^. However, the direct role of SREBP-1c for iron regulation in HSCs is unknown. The role of LCN2 in hepatocytes has been described by Xu et al., which stated the beneficial effects of LCN2, including protection against diet-induced NAFLD through the regulation of lipolysis and fatty acid oxidation and the inhibition of de novo lipogenesis^49^. This study revealed the role of SREBP-1c in the regulation of LCN2 expression, which is involved in iron transport and sequestration. In addition, CCl_4_-induced NASH was associated with increases in SREBP-1c and LCN2 levels, while 1cKO mice exhibit decreased LCN2 expression, suggesting that SREBP-1c and other factors regulate CCl_4_-induced NASH.

Overall, this study confirmed that decreasing LCN2 ameliorated liver fibrosis by regulating intracellular iron levels in HSCs. The SREBP-1c-LCN2-Fe^3+^-SMAD axis is a new mechanism of hepatocyte-HSCs communication in chronic NASH. The evidence suggests that SREBP-1c can regulate intracellular iron levels through its effects on genes involved in iron metabolism and storage; this regulation may be important for treating metabolic disorders, inflammation, and other pathological conditions. However, further studies are needed to fully elucidate the mechanisms by which SREBP-1c regulates intracellular iron and determine its importance in various physiological and pathological contexts.

### Supplementary information

Supplementary information
