# Supplementary material for: Loss of SREBP-1c ameliorates iron-induced liver fibrosis by decreasing lipocalin-2
Source: Exp Mol Med. 2024 Apr 16;56(4):1001–12. doi: 10.1038/s12276-024-01213-2 (PMC11058876; doi:10.1038/s12276-024-01213-2)

SUPPORTING INFORMATION

Loss of SREBP-1c ameliorates iron-induced liver fibrosis by decreasing lipocalin-2

Eun-Ho Lee, Jae-Ho Lee, Do-Young Kim, Young-Seung Lee, Yunju Jo, Tam Dao, Kyung Eun Kim, Dae-Kyu Song, Ji Hae Seo, Young-Kyo Seo, Je-Kyung Seong, Changjong Moon, Eugene Han, Mi Kyung Kim, Seungwan Ryu, Minsang Shin, Gu Seob Roh, Hye Ra Jung, Timothy F. Osborne, Dongryeol Ryu, Tae-Il Jeon, Seung-Soon Im

SUPPLEMENTARY MATERIALS AND METHODS

Animal studies

Male 8-week-old WT and LCN2 KO littermates were fed with either a chow diet or a high-fat diet (HFD, D12492, 60% fat, Research Diets, NJ, USA) containing for 20 weeks (n = 5 per group).

Mouse primary hepatocytes isolation

Primary hepatocytes were extracted as previously reported [1]. Mouse primary hepatocytes were isolated from 10-week-old male WT and 1cKO mice by collagenase perfusion. Under the tissue culture hood, the minced liver capsule was taken with forceps and carefully shaken until the hepatocytes were released. The hepatocyte suspension was plated on rat tail collagen (COL) type I-coated plates in an attachment medium (William's E medium supplemented with 10% fetal bovine serum (FBS), 1% GlutaMax, and 1% P/S). After allowing the cells to attach for 4 h, they were washed and further incubated in high-glucose DMEM medium supplemented with 10% FBS and 1% P/S.

Mouse primary HSCs and Kupffer cells isolation

HSCs and Kupffer cells were isolated from WT and 1cKO mice as described previously. Briefly, livers were perfused in situ through inferior vena cava warm perfusion HBSS solution. The enzymatic treatment was carried out in the genetleMACS processor at 37 °C for 30 min with constant shaking using the m liver 03 program. HSCs were collected between the HBSS layer and 11.5% optiprep layer and Kupffer cells were collected between the 11.5% layer and 17.5% optiprep layer. HSCs and kupffer cells were cultured in Dulbecco's modified Eagle's medium (DMEM, HyClone, Logan, UT, USA) supplemented

with 10% FBS (HyClone) and 1% penicillin/streptomycin (P/S, Gibco, Invitrogen, Carlsbad, CA, USA) respectively and kupffer cells were plated with an extra supplement of 20% L929 for 5 days.

Promoter activity

The mouse LCN2 reporter (pGL3-mLCN2) was provided by Dr. Don-Kyu Kim (Chonnam National University, Republic of Korea). The ΔSRE reporter was constructed by deleting the SRE (5'-GTGGGGTCA-3') motif by using the site-directed mutagenesis kit (Agilent Technologies, Santa Clara, CA). 2×flag pCDNA3.1+SREBP-1c has been previously described. All constructs were verified by DNA sequencing. The cells were transfected with luciferase reporters and expression plasmids using Lipofectamine 2000 (Invitrogen, Waltham, MA). A pCMV-β-gal expression construct was included in every transfection as a normalization control. After 24 h post-transfection, cells were harvested, and luciferase activity was measured using a EZ Luciferase assay system (Enzynomics, Republic of Korea).

Transfection of siRNA

siRNA targets of human SREBF1 (M-006891-01-0010, Dharmacon, Lafayette, CO, USA) were purchased. Cells were transfected with SREBF1 siRNA and control scrambled siRNA using lipofectamine RNAiMAX (13778-075, Invitrogen) following the manufacturer's instructions.

Preparation of holo-LCN2 conjugated with enterobactin

For holo-LCN2 treatment, 250 μM enterobactin (E3910, Sigma-Aldrich, St Louis. MO, USA), 250 μM iron (III) chloride hexahydrate (236489, Sigma-Aldrich), and 24 ng/μl LCN2 protein

(provided by Dr. M.-S. Shin, kyungpook National University, Korea) were mixed. After incubation in the dark for 75 min, centrifugation (10,000 g, 4°C) was performed 3 times for 65 min each with PBS using a YM-10 tube (MRCPRT010, Merck Millipore, Billerica, MA, USA). The prepared holo-LCN2 was quantified by BCA assay (Thermo Fisher Scientific, Waltham, MA, USA) and the concentration was confirmed before use.

Preparation of fatty acid solutions

For FA treatment, bovine serum albumin (BSA; A3803, Sigma-Aldrich)-conjugated palmitic acid (PA; P0500, Sigma-Aldrich) and oleic acid (OA; O3008, Sigma-Aldrich) were prepared using a modified method described previously [2]. First, PA was dissolved in 100% ethyl alcohol at 7.5 mM, and BSA was dissolved in serum-free DMEM. A final concentration of 200 μM PA or 100 μM OA conjugated with BSA was achieved through further dilutions.

ChIP assay

Samples for ChIP assays using mouse tissues were prepared as described previously [3]. Brief procedures were followed. Freshly isolated livers from 4 mice/fasting after refeeding group were pooled and disrupted in final concentration of 1% formaldehyde containing a mixture of protease inhibitors (1 mg/ml leupeptin, 1.4 mg/ml pepstatin, 2 mg/ml phenylmethylsulfonyl fluoride, and 1 mM of EGTA). Final DNA samples were analyzed for gene-specific ChIP. Chromatin size was checked by agarose electrophoresis to ensure that the average size was between 200 and 500 base pairs. Aliquots were then used for chromatin immunoprecipitation assay with antibodies specific to SREBP-1 as described previously [4]. For gene-specific ChIP, qPCRs for SREBP-1 binding to specific gene promoters, *Fas*, *Ldlr*, *Lcn2*, were analyzed in triplicate with a standard dilution curve of the input DNA performed in parallel, and

enrichment was measured by SYBR green incorporation using an iCycler (Bio-Rad, Hercules, CA, USA). Analysis was performed by the standard curve method, and values were normalized to a nontarget control region from the ribosomal *L32* gene. The fold change is the fold increase for the signal from SREBP-1-antibody-enriched chromatin, relative to a control IgG fraction.

RNA isolation and qRT-PCR

Total RNA was isolated from the cell and mouse livers with TRIzol Reagent (Invitrogen) and reverse-transcribed using the cDNA iScript kit (Bio-Rad). qRT-PCR was performed using the CFX96 Bio-Rad qRT-PCR machine (Bio-Rad). PCR amplifications were subjected using iQ™ SYBR Green Supermix (Bio-Rad) with specific primers (Supplementary Table 2). mRNA levels were normalized for expression of *L32* and *RPLP0* as control and calculated by the comparative threshold cycle method. All samples were run in triplicate, and average values were calculated.

Western blot analysis

Proteins were isolated from cells and mouse livers was performed following a modified protocol of previous method [5]. For protein extraction, cells and frozen livers were homogenized in RIPA and T-PER lysis buffer (Thermo Fisher Scientific) with a protease and phosphatase inhibitor cocktail (Thermo Fisher Scientific). Protein concentration was measured by a BCA assay (Thermo Fisher Scientific). Proteins were resolved by SDS polyacrylamide gel electrophoresis, transferred to 0.2 mm nitrocellulose membranes (GE Healthcare-Amersham, Chicago, IL, USA) by wet transfer at 4 °C, washed in tris-buffered Saline containing tween 20 (TBST) for 30 min, blocked in 5% skim milk for 1 h at room

temperature, and washed for a further 30 min in TBST prior to incubation with primary antibodies. The primary antibodies used are shown in Supplementary Table 3. β-actin and GAPDH were used as loading controls to normalize protein levels in total fractions. Protein bands were detected using ECL (Bio-Rad), and chemiluminescence was analyzed using a Fusion Fx imaging system (Vilber Lourmat, Collégien, France).

Histological staining

Liver specimens were fixed in 4% paraformaldehyde (pc2031-100-00, Biosesang, Seongnam, Korea), embedded in paraffin, and cut into 4 μm-thick sections. Sections were dewaxed, hydrated, and stained per standard protocols with hematoxylin and eosin (H&E; BBC Biochemical, Mt. Vernon, WA, USA), Masson's trichrome (BBC Biochemical), Sirius red (ab246832, Abcam, Cambridge, UK), LCN2 (AF1857, R&D Systems, Minneapolis, MN, USA), α-SMA (A5228, Sigma-Aldrich), and TUNEL staining (Promega, Madison, WI, USA).

ELISA

Serum triglycerides (TG) and cholesterol were extracted using McGowan's [6] and Richmond's [7] method as described previously. Serum TG levels were quantified using a TG assay kit (AM 157S-K, Asan Pharm Co., Seoul, Korea), serum cholesterol levels were quantified using a total cholesterol assay kit (AM 202-K, Asan Pharm Co.), and the absorbance of the samples and standard was measured at 500 or 550 nm using Infinite® 200 PRO (Tecan Trading AG, Männedorf, Switzerland). Hepatic injury was determined by measuring levels of serum aspartate aminotransferase (AST) and alanine aminotransferase (ALT) using spectrophotometric assay kits (AM 102-K and AM 103-K, Asan Pharm Co.). The absorbance values of serum biochemicals were measured at a wavelength of 505 nm. The lipid

peroxidation product MDA concentration in cell lysates was assessed using a Lipid Peroxidation Assay Kit (ab118970, Abcam) according to the manufacturer’s instructions. The relative iron concentration in cell lysates was assessed using an Iron Assay Kit (MAK025-1kt, Sigma-Aldrich) according to the manufacturer’s instructions. The cell culture supernatants were upon centrifugation for 10 min at 12,000 rpm at 4 °C. The LCN2 level in cell culture supernatants and serum were quantified following the manufacturer’s protocols for lipocalin-2/NGAL Quantikine ELISA kit (Cat# MLCN20 and DLCN20, R&D systems).

Flow cytometry analysis

The apoptosis assay was performed using Annexin V/ PI method, following the manufacturer's instructions (FITC Annexin V apoptosis detection kit I, BD Biosciences, San Jose, CA, USA). After treatment, the harvested cells were washed in cold PBS, resuspended in 100 μl of binding buffer (V13242, Invitrogen), and stained with 5 μl FITC Annexin V (556419, BD Biosciences) and PI (P4170, Sigma-Aldrich) for 15 min at room temperature in the dark. Add 400 μl of binding buffer to each tube. Apoptotic cells were analyzed by BD FACS Caliber (BD Biosciences).

Bioinformatic analysis

All data, including transcriptomes and hematoxylin and eosin (H&E)-stained histological images, that were analyzed in this study are available at the Genotype-Tissue Expression (GTEx) portal (data ver. 8, https://www.gtexportal.org) and the Gene Expression Omnibus (GEO) (https://www.ncbi.nlm.nih.gov/geo/; access ID: GSE135251) of the National Center for Biotechnology Information. Gene set enrichment analysis (GSEA) was performed as

previously described (Pmid 34854561) GSEA-enrichment plots including NES (x axis), nominal p-values (bubble color), and gene set size (bubble size) were generated with GSEA v4.2.3 for Windows. Visualization of bioinformatic analyses was conducted with RStudio (RStudio Desktop 2022.02.3 Build 492; R 4.2.1), installed R packages corrr, dplyr, stringr, ggpubr, ggplot2, pheatmap, egg, reshape2, gridExtra, and RColorBrewer (https://www.r-project.org) as described previously [8,9].

Supplementary Tables

Supplementary Table 1. Physical and biochemical parameters associated with the normal and NASH patients

Characteristics	Normal	NASH
No. (male/female)	36 (36/0)	35 (14/21)
Age (years), median (range)	38 (30-50)	40 (25-67)
BMI (kg/m^2), median (range)	21.9 (20-30)	38.6 (28-56.1)
FBS (mg/dL), median (range)	90 (77-98)	109 (74-184)
HbA1c (%), median (range)	5.3 (4.6-5.6)	6.2 (4.3-10.3)
TC (mg/dL), median (range)	169 (132-214)	186 (94-257)
TG (mg/dL), median (range)	96 (13-282)	178 (55-717)
HDL (mg/dL), median (range)	49 (35-73)	44 (29-79)
LDL (mg/dL), median (range)	97 (51-139)	119 (38-185)
AST (U/l), median (range)	19 (10-34)	37 (13-193)
ALT (U/l), median (range)	15 (6-44)	47 (12-157)

ALT, alanine aminotransferase; AST, aspartate aminotransferase; BMI, body mass index; FBS, Fasting blood sugar; HbA1c, glycated hemoglobin; HDL, high density lipoprotein; LDL, low density lipoprotein; NASH, non-alcoholic steatohepatitis; TC, total cholesterol; TG, triglyceride.

Supplementary Table 2. Primer sequences used for qPCR

Gene	Forward primer	Reverse primer
α-sma	GTTCAGTGGTGCCTCTGTCA	ACTGGGACGACATGGAAAAG
Col1α1	TTCGGACTAGACATTGG	GGGTTGTTCGTCTGTTTC
Col3α1	ACGTAGATGAATTGGGATGCAG	GGGTTGGGGCAGTCTAGTG
Col5α2	TTGGAAACCTTCTCCATGTCAGA	TCCCCAGTGGGTGTTATAGGA
Col6α1	CTGCTGCTACAAGCCTGCT	GCACGAAGAATAGATCCACAGGG
Tnf-α	CCCTCACACTCAGATCATCTTCT	GCTACGACGTGGGCTACAG
Srebp-1c	GGAGCCATGGATTGCACATT	GCTTCCAGAGAGGAGGCCAG
Fas	AGCTTCGGCTGCTGTTGGAAGT	TCGGATGCCTCTGAACCACTCACA
Acc1	TGACAGACTGATCGCAGAGAAAG	TGGAGAGCCCCACACACA
Scd1	CCGGAGACCCCTTAGATCGA	TAGCCTGTAAAAGATTTCTGCAAACC
Mcp1	CCACTCACCTGCTGCTACTCA	TGGTGATCCTCTTGTAGCTCTCC
Cd36	GAACAGCAGCAAAATCAAGG	TCCAACAGACAGTGAAGGC
Pparα	AGAGCCCCATCTGTCCTCTC	ACTGGTAGTCTGCAAAACCAAA
Acc2	GGGCTCCCTGGATGACAAC	GCTCTTCCGGGAGGAGTTCT
Tgf-β1	ATTTGGAGCCTGGACACACA	GAGCGCACAATCATGTTGGA
Timp1	GAGACCACCTTATACCAGCGTT	TACGCCAGGGAACCAAGAAG
Mmp2	ACCTGAACACTTTCTATGGCTG	CTTCCGCATGGTCTCGATG
Mmp9	GCGTCGTGATCCCCACTTAC	CAGGCCGAATAGGAGCGTC
Pai1	TTCAGCCCTTGCTTGCCTC	ACACTTTTACTCCGAAGTCGGT
Lcn2	TGGCCCTGAGTGTCATGTG	CTCTTGTAGCTCATAGATGGTGC
Il-6	GTGACGTTGACATCCGTAAAGA	GCCGGACTCATCGTACTCC
F4/80	CTTTGGCTATGGGCTTCCAGTC	GCAAGGAGGACAGAGTTTATCGTG
Il-1β	CCGTGGACCTTCCAGGATGA	GGGAACGTCACACACCAGCA
Des	ATGTCCAAGCCAGACCTCAC	AGGCCATCTTCACATTGAGC
24p3r	GATAGACAGGAAGGCAAGGC	GACGGAGTGAACAGAAAGCA
L32	ACATTTGCCCTGAATGTGGT	ATCCTCTTGCCCTGATCCTT
LCN2	CAAGGAGCTGACTTCGGAAC	GACAGGGAAGACGATGTGGT
SREBP-1c	ACACAGCAACCAGAAACTCAAG	AGTGTGTCCTCCACCTCAGTCT
α-SMA	CTGGCATCGTGCTGGACTCT	GATCTCGGCCAGCCAGATC
TGF-β1	CAATTCCTGGCGATACCTCAG	GCACAACTCCGGTGACATCAA
MMP2	CCCACTGCGGTTTTCTCGAAT	CAAAGGGGTATCCATCGCCAT

MMP9	TGTACCGCTATGGTTACACTCG	GGCAGGGACAGTTGCTTCT
COL1α1	GTGCGATGACGTGATCTGTGA	CGGTGGTTTCTTGGTCGGT
COL3α1	GGAGCTGGCTACTTCTCGC	GGGAACATCCTCCTTCAACAG
COL5α2	GACTGTGCCGACCCTGTAAC	CCTGGACGACCACGTATGC
COL6α1	ACACCGACTGCGCTATCAAG	CGGTCACCACAATCAGGTACTT
F4/80	GCTGTGATACTGTTCTTGATGGT	CAGCATCGGCAGCCCATAA
TNF-α	GAGGCCAAGCCCTGGTATG	CGGGCCGATTGATCTCAGC
IL-1β	TTCGACACATGGGATAACGAGG	TTTTTGCTGTGAGTCCCGGAG
MCP1	CAGCCAGATGCAATCAATGCC	TGGAATCCTGAACCCACTTCT
RPLP0	GTGCTGATGGGCAAGAAC	AGGTCCTCCTTGGTGAAC
ChIP-Ldlr	GAACTTCCCACTGCTGC	CACGCCCAGAGTCATTC
ChIP-Fas	GCGCAGCCCCGACGCTCATT	CGGCGCTATTTAAACCGCGG
ChIP-Lcn2	CTGCCAGAATCCAAAGCCCTG	AGTCCAGGAAGCCATG

These primer sequences were used for qRT-PCR. The expression level of mRNA was normalized by the expression level of L32 and RPLP0 mRNA.

Supplementary Table 3. List of primary antibodies used in this study

Antibody	Company	Catalog No.	Source
LCN2	Abcam	ab63929	Rabbit
LCN2	R&D Systems	AF1857	Mouse
SREBP1	Santa Cruz	SC-13551X	Mouse
COL1A1	CST	91144T	Rabbit
α-SMA	CST	D4K9N	Rabbit
TGF-β1	CST	3709T	Rabbit
FAS	CST	3180	Rabbit
ACC1	CST	4190	Rabbit
SCD1	CST	2794	Rabbit
TGFBR2	CST	79424T	Rabbit
SMAD2	CST	5339T	Rabbit
p-SMAD2	CST	3108T	Rabbit
SMAD4	CST	38454T	Rabbit
SMAD7	Santa Cruz	sc-101152	Mouse
MMP9	Sigma-Aldrich	M9555	Rabbit
TIMP1	Santa Cruz	sc-21734	Mouse
PARP	CST	9542	Rabbit
β-ACTIN	Sigma-Aldrich	A5441	Mouse
GAPDH	CST	2118	Rabbit

Supplementary references

1. Pantha, R. et al. Perilipin 5 is a novel target of nuclear receptor LRH-1 to regulate hepatic triglycerides metabolism. *BMB Rep*. **54**, 476-481 (2021).
2. Lennon, R. et al. Saturated fatty acids induce insulin resistance in human podocytes: implications for diabetic nephropathy. *Nephrol Dial Transplant*. **24**, 3288-3296 (2009).
3. Bennett, M. K., Seo, Y. K., Datta, S., Shin, D. J. & Osborne, T. F. Selective binding of sterol regulatory element-binding protein isoforms and co-regulatory proteins to promoters for lipid metabolic genes in liver. *J Biol Chem*. **283**, 15628-15637 (2008).
4. Seo, Y. K. et al. Genome-wide analysis of SREBP-1 binding in mouse liver chromatin reveals a preference for promoter proximal binding to a new motif. *Proc Natl Acad Sci U S A*. **106**, 13765-13769 (2009).
5. Im, S. S. & Osborne, T. F. Protection from bacterial-toxin-induced apoptosis in macrophages requires the lipogenic transcription factor sterol regulatory element binding protein 1a. *Mol Cell Biol*. **32**, 2196-2202 (2012).
6. McGowan, M. W., Artiss, J. D., Strandbergh, D. R. & Zak, B. A peroxidase-coupled method for the colorimetric determination of serum triglycerides. *Clin Chem*. **29**, 538-542 (1983).
7. Richmond, W. Use of cholesterol oxidase for assay of total and free cholesterol in serum by continuous-flow analysis. *Clin Chem*. **22**, 1579-1588 (1976).
8. Chung, H. et al. Artificial-intelligence-driven discovery of prognostic biomarker for sarcopenia. *J Cachexia Sarcopenia Muscle*. **12**, 2220-2230 (2021).
9. Ji, M. et al. Plasma Metabolomics and Machine Learning-Driven Novel Diagnostic Signature for Non-Alcoholic Steatohepatitis. *Biomedicines*. **10**, (2022).

Supplementary Figures

Supplemental Fig. 1 NASH development of HFHS-fed SREBP-1cKO mice. WT and 1cKO mice (n = 5-10 per group) were fed either CD or HFHS diets for 20 weeks. **a** Food consumption in each group over a period of 20 weeks. **b**, **c** Fat, and lean body mass were normalized by body weight. **d** Serum levels of AST, ALT, TG, and TC were measured in CD or HFHS-fed WT and 1cKO. **e** qPCR analysis of *Srebp-1c*, *Fas*, *Acc1*, and *Scd1* mRNA expression levels in the livers of WT and 1cKO mice after a CD or a HFHS diets feeding. Relative mRNA expression was normalized by *L32* expression level. **f** Western blot analysis for determining FAS, ACC1, and SCD1 protein levels. Protein samples were prepared from WT and 1cKO mice livers. **g-k** The hepatic mRNA levels of fibrogenesis genes such as *Col3α1, Col5α2, Mcp1, Cd36, Pparα,* and *Acc2*. Values are expressed as mean ± SEM. $^{*}p < 0.05$, $^{**}p < 0.01$, and $^{***}p < 0.001$ compared to Con WT mice. $^{\#}p < 0.05$, and $^{\#\#}p < 0.01$ compared to HFHS-fed treated WT mice.

Supplemental Fig. 2 Liver fibrosis of CCl_4-treated 1cKO mice. WT and 1cKO mice (n = 3-5 per group) were treated with PBS and CCl_4 treated for 5 weeks. (**a** and **b**) Fat, and lean body mass were normalized by body weight. **c** The weight of liver tissues. **d** Liver sections were stained with H&E (scale bars: 100, and 30 μm). **e** Serum levels of AST, and ALT were measured in CCl_4-treated WT and 1cKO mice. **f**, **g** The hepatic mRNA levels of lipogenic and fibrogenic genes such as *Srebp-1c, Fas, Mmp9, Col6α1, Timp1,* and *Pai1*. **h** Western blot analysis for determining pSREBP-1, nSREBP-1, α-SMA, MMP9, COL1α1, TIMP1, TGF-β1, TGFβR2, p-SMAD2, SMAD2, SMAD4, and SMAD7 protein levels. Protein samples were prepared from CCl_4-treated WT and 1cKO mice livers. **i** mRNA levels of *F4/80*, *Tnf-α*, *Il-1β*,

Il-6, and *Mcp1* were measured using qPCR. Values are expressed as mean ± SEM. $^{*}p < 0.05$, $^{**}p < 0.01$, and $^{***}p < 0.001$ compared to Mock WT mice. $^{\#}p < 0.05$, and $^{\#\#}p < 0.01$ compared to CCl_4 treated WT mice.

Supplemental Fig. 3 Effect of LCN2 gene expression by CCl_4-treated 1cKO mice on primary kupffer cells. WT and 1cKO mice (n =3-5 per group) were treated with PBS and CCl_4 treated for 5 weeks. **a** *Lcn2* mRNA expression levels in primary kupffer cells isolated from CCl_4-treated WT and 1cKO mice. **b** Immunoblotting for LCN2 protein level in kupffer cells. **c** Analysis of LCN2 concentration in kupffer cells. **d** qPCR analysis of *Srebp-1c*, *Fas*, *Acc1*, and *Scd1* mRNA expression in above samples. **e** mRNA expression level of *Tgf-β1* gene. **f** TGF-β1 protein level in kupffer cells. **g** *F4/80*, *Tnf-α*, *Il-1β*, *Il-6*, and *Mcp1* mRNA levels in kupffer cells. Values are expressed as mean ± SEM. $^{*}p < 0.05$, $^{**}p < 0.01$, and $^{***}p < 0.001$ compared to Mock WT mice. $^{\#}p < 0.05$, $^{\#\#}p < 0.01$, and $^{\#\#\#}p < 0.001$ compared to CCl_4 treated WT mice.

Supplemental Fig. 4 Effects of FA on ferroptosis in mouse primary hepatocytes. a-c The hepatic mRNA levels of ferroptosis genes such as *Ncoa4*, *Ptgs2*, *Fth1*, *Slc7a11*, *Acsl4*, *Nfe2l2*, *Hmox1*, *Slc11a2*, and *Tfrc* measured in primary hepatocytes from WT and 1cKO mice. **d** Measurement of LCN2 concentration in cells after FA treatment transfected with siRNA targeting SREBP-1i. Values are expressed as mean ± SEM.

Supplemental Fig. 5 Effects of apo-LCN2, and holo-LCN2 on apoptosis. a Iron-induced SMAD-dependent TGF-β signaling pathways. **b** PARP, cleaved PARP protein level in HSCs. **c**, **d** Representative flow cytometry histograms showing Annexin V-FITC population. Quantitative analyses of mock, apo-LCN2, and holo-LCN2.

Supplemental Fig. 6 HFD-induced hepatic steatosis and fibrosis are reduced in LCN2 LKO mice. WT and LCN2 KO mice were CD or HFD for 20 weeks to induce obesity (n = 5 per group). **a** Representative images of liver sections stained with H&E, Sirius Red, and α-SMA (scale bars: 100, and 50 μm). **b**, **c** Western blot analysis and quantification of LCN2, and α-SMA protein levels in WT and LCN2 KO mice livers. Values are expressed as mean ± SEM. $^{*}p < 0.05$, and $^{**}p < 0.01$ compared to Con WT mice. $^{\#}p < 0.05$ compared to HFD WT mice.

Supplemental Fig. 7 LCN2 overexpression in CCl_4 treated 1cKO mice. WT and 1cKO mice (n = 5-7 per group) were treated with CCl_4 (5 weeks) and adenoviruses in mice groups. **a** Body weight, increased body weight, fat, and lean body mass were normalized by body weight. **b** Liver weight was normalized by body weight. **c** The hepatic mRNA levels of *Srebp-1c*. qPCR analysis of *Srebp-1c* mRNA expression levels in livers. **d-h** The mRNA levels of *Timp1*, *Tgf-β1*, *Pai1*, *Des*, and *24p3r*. Values are expressed as mean ± SEM. $^{*}p < 0.05$ compared to CCl_4 treated WT mice. $^{\#}p < 0.05$ compared to CCl_4 treated 1cKO mice.

Supplemental Fig. 8 Expression levels of fibrosis and inflammation marker genes in human liver. a, b qPCR analysis of *α-SMA*, *TGF-β1*, *MMP2*, *MMP9*, *COL1α1*, *COL3α1*,

COL5α2, *COL6α1*, *F4/80*, *TNF-α*, *IL-1β*, and *MCP1* mRNA expression from the normal humans and NASH patients' liver. Values are expressed as mean ± SEM. $^{***}p < 0.001$ compared to Normal.

Supplementary Figures

Supplementary Fig. 1.

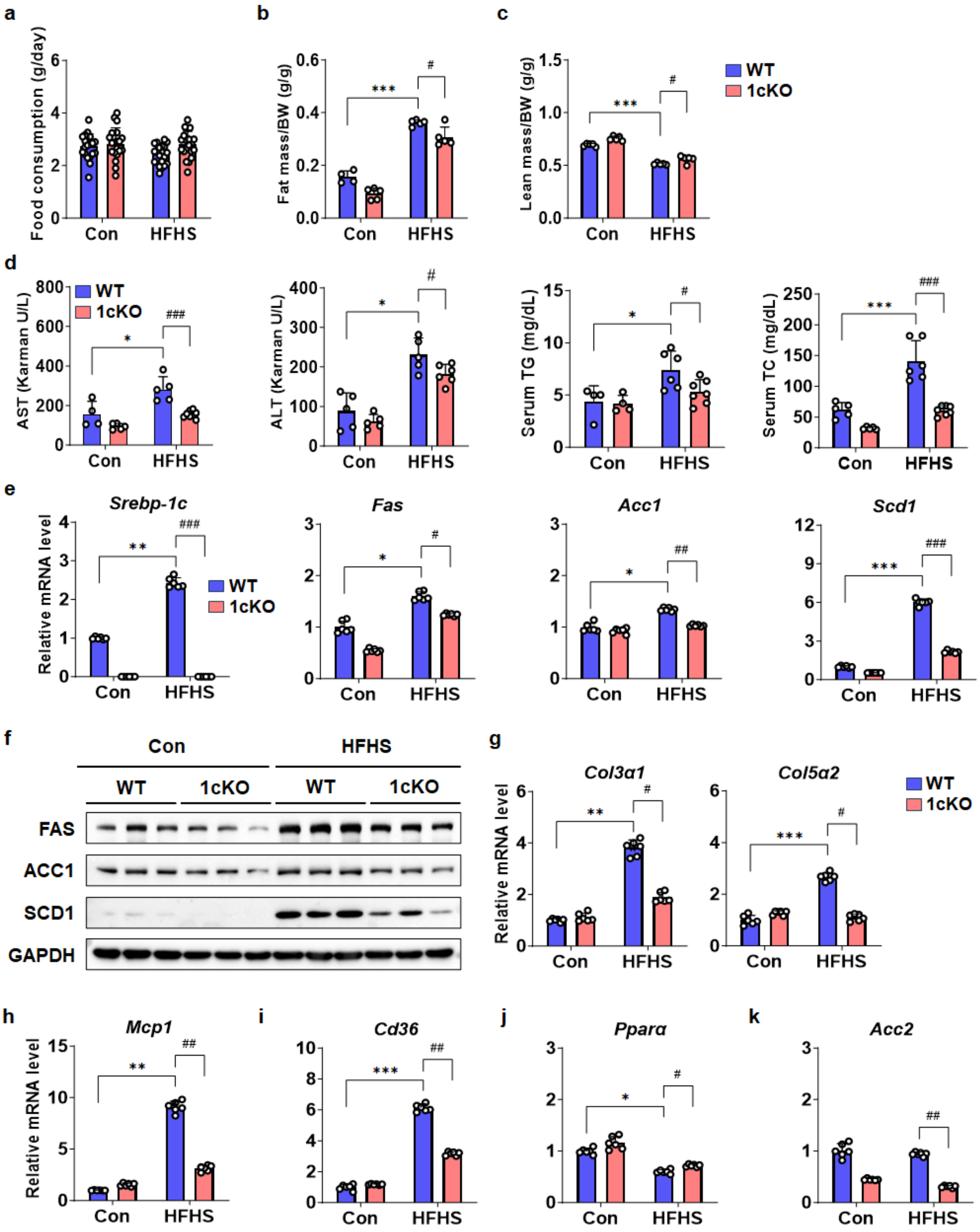

Supplementary Fig. 2.

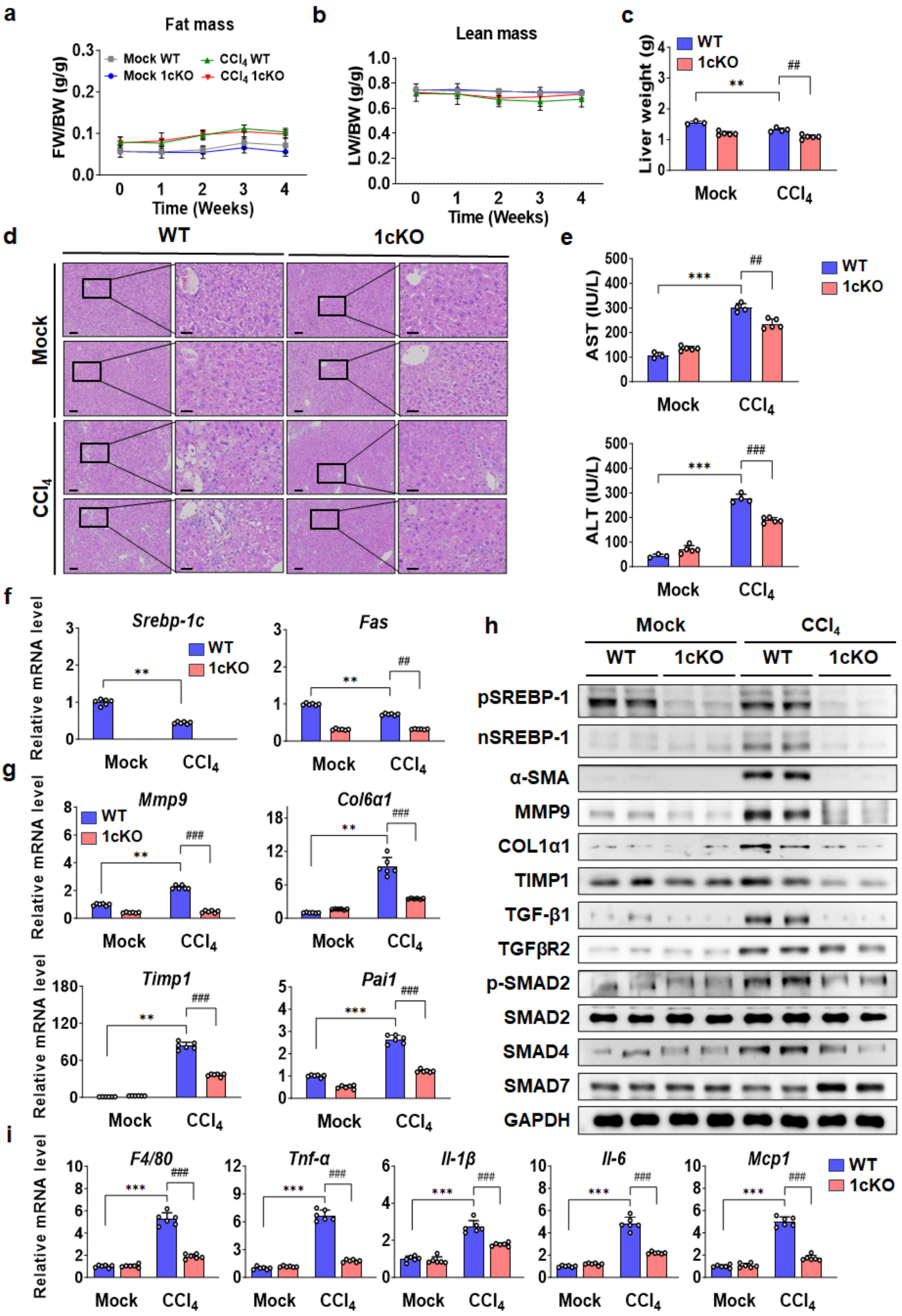

Supplementary Fig. 3.

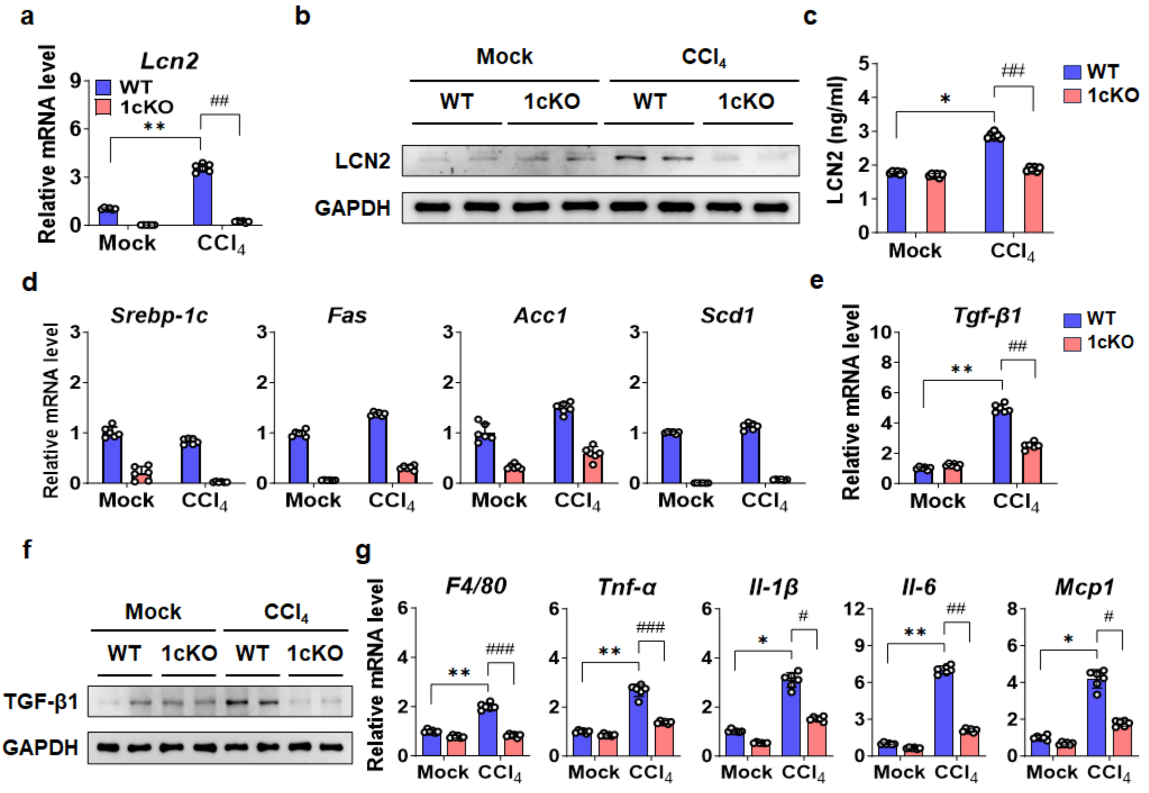

Supplementary Fig. 4.

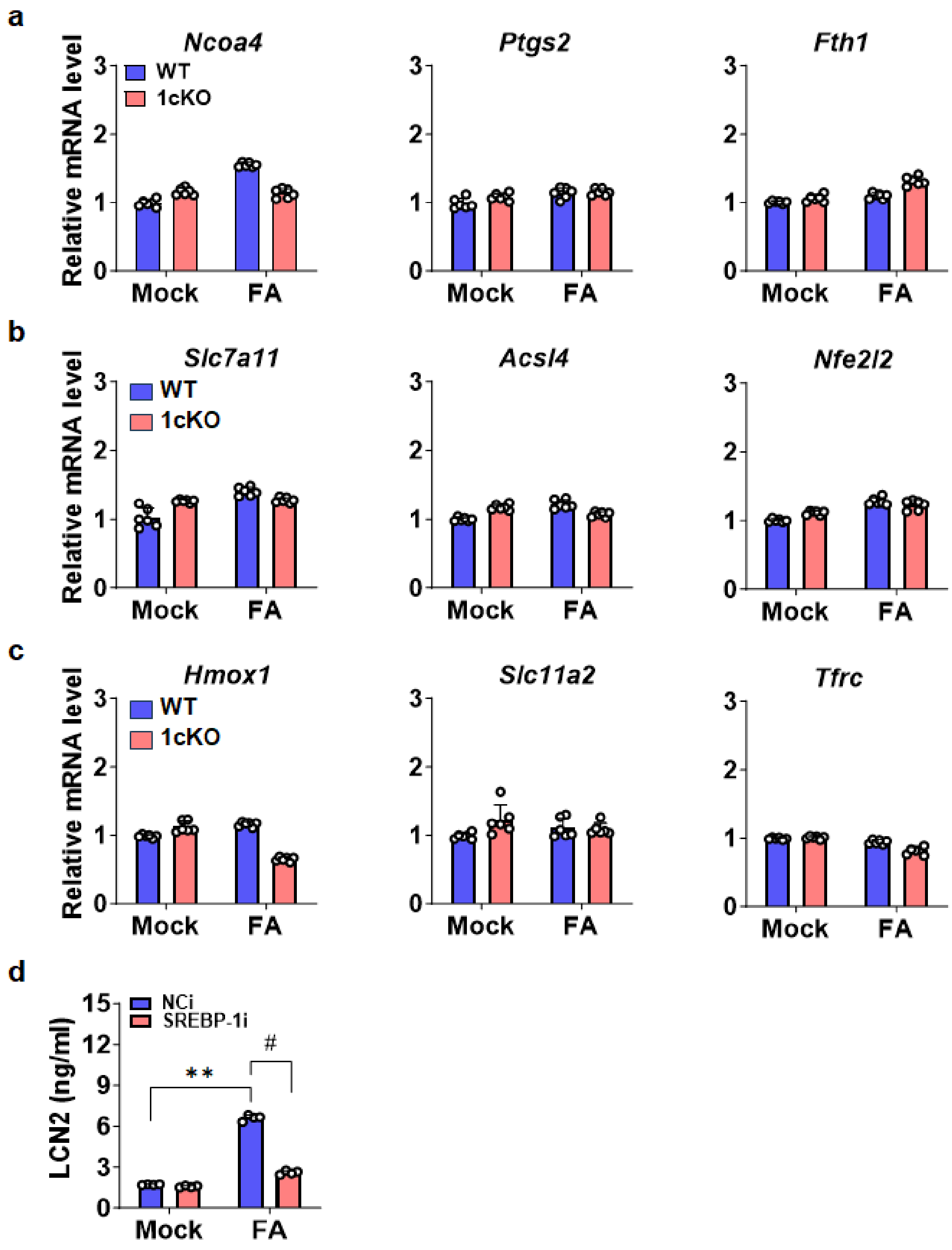

Supplementary Fig. 5.

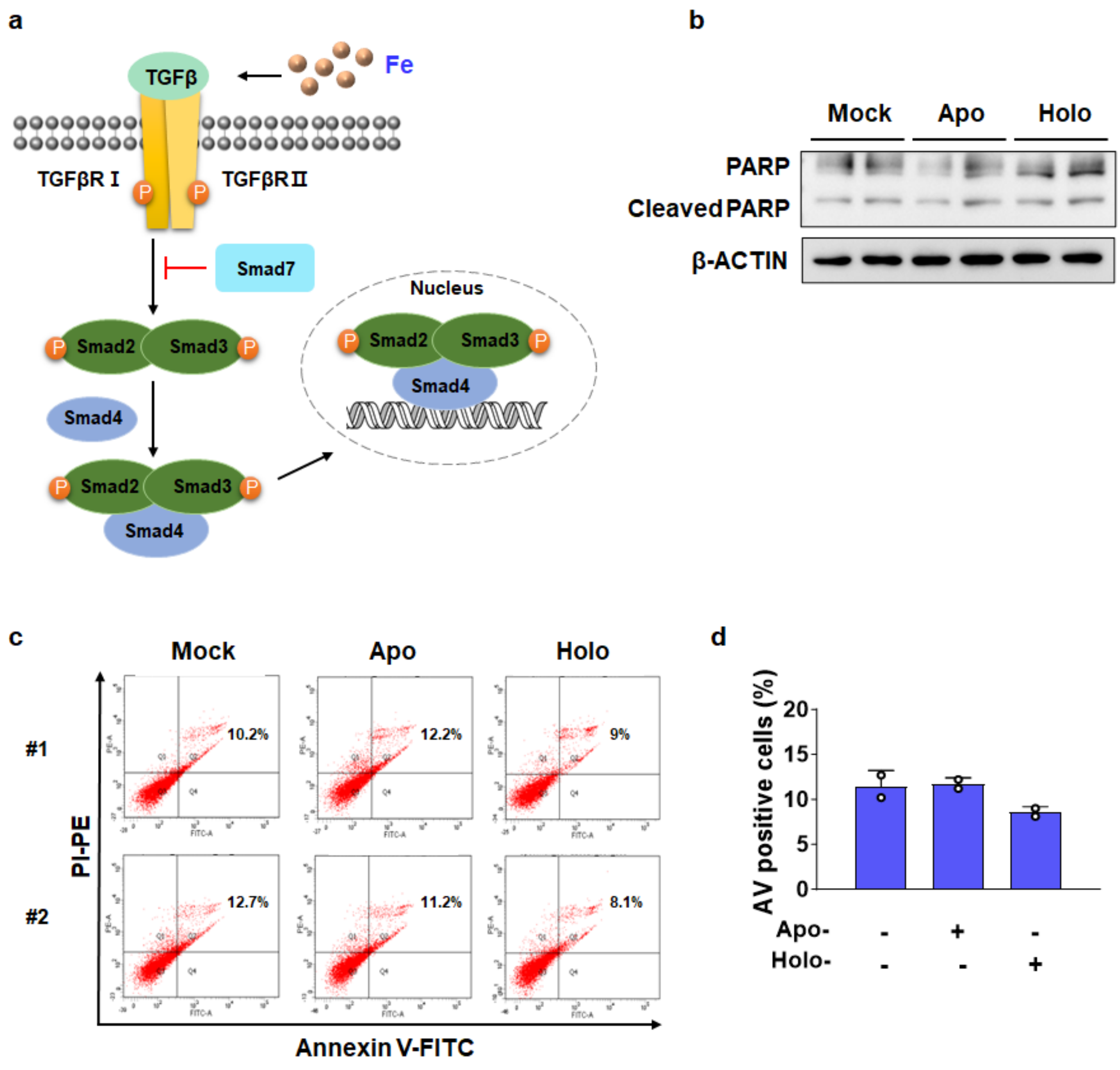

Supplementary Fig. 6.

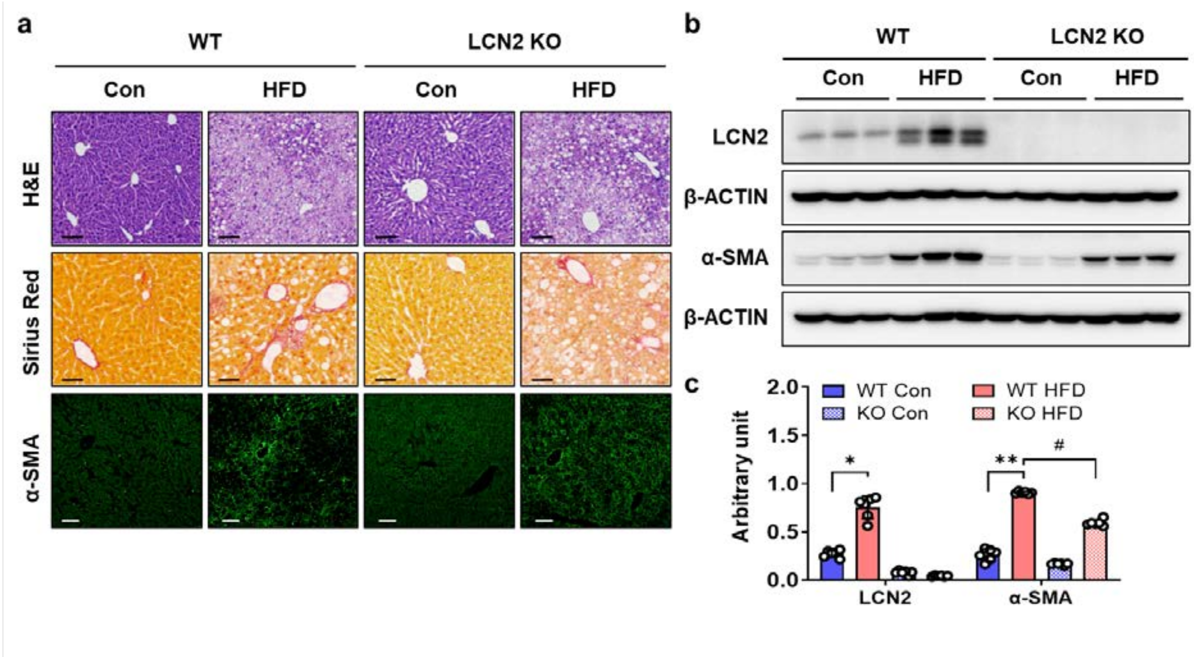

Supplementary Fig. 7.

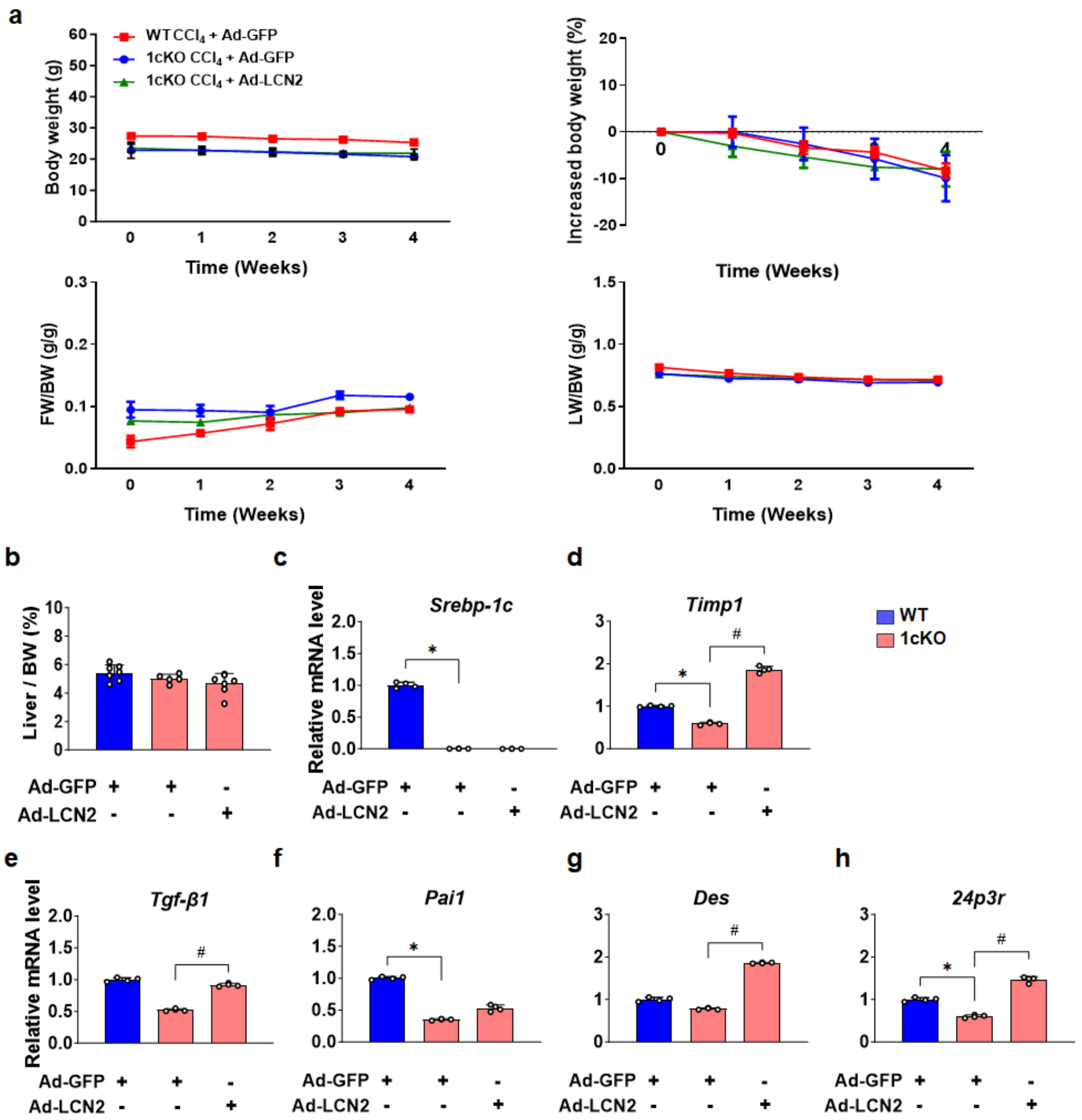

Supplementary Fig. 8.

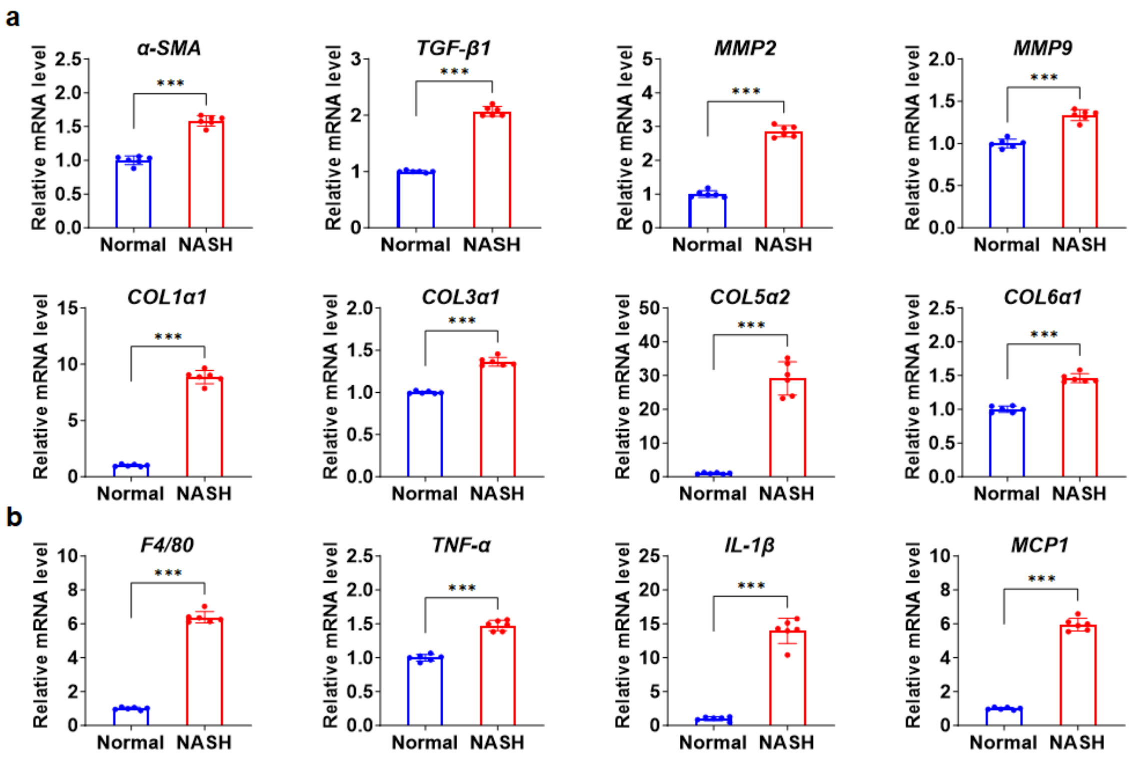

Supplement: Supplementary file 1 — Supplementary information [file 12276_2024_1213_MOESM1_ESM.pdf]
